# Investigating the physical activity, health, wellbeing, social and environmental effects of a new urban greenway: a natural experiment (the PARC study)

**DOI:** 10.1186/s12966-021-01213-9

**Published:** 2021-10-30

**Authors:** Ruth F. Hunter, Deepti Adlakha, Christopher Cardwell, Margaret E. Cupples, Michael Donnelly, Geraint Ellis, Aisling Gough, George Hutchinson, Therese Kearney, Alberto Longo, Lindsay Prior, Helen McAneney, Sara Ferguson, Brian Johnston, Michael Stevenson, Frank Kee, Mark A. Tully

**Affiliations:** 1 Centre for Public Health, Queen’s University Belfast, Institute of Clinical Sciences B, Royal Victoria Hospital, Grosvenor Road, Belfast, BT12 6BJ Northern Ireland, UK; 2 Department of Landscape Architecture and Environmental Planning, Natural Learning Initiative, College of Design, North Carolina State University, Raleigh, NC 27607 USA; 3 School of Natural and Built Environment, Queen’s University Belfast, Belfast, Northern Ireland; 4 School of Nursing, Queen’s University Belfast, Belfast, Northern Ireland; 5 Gibson Institute and Institute for Global Food Security, School of Biological Sciences, Queen’s University Belfast, Belfast, Northern Ireland; 6 Institute of Mental Health Sciences, School of Health Sciences, Ulster University, Newtownabbey, Northern Ireland

**Keywords:** Urban green space, Intervention, Natural experiment, Physical activity, Health, Mental wellbeing

## Abstract

**Background:**

Evidence for the health benefits of urban green space tends to stem from small, short-term quasi-experimental or cross-sectional observational research, whilst evidence from intervention studies is sparse. The development of an urban greenway (9 km running along 3 rivers) in Northern Ireland provided the opportunity to conduct a natural experiment. This study investigated the public health impact of the urban greenway on a range of physical activity, health, wellbeing, social, and perceptions of the environment outcomes.

**Methods:**

A repeated cross-sectional household survey of adult residents (aged ≥16 years) who lived ≤1-mile radius of the greenway (intervention sample) and > 1-mile radius of the greenway (control sample) was conducted pre (2010/2011) and 6-months post implementation (2016/2017). We assessed changes in outcomes pre- and post-intervention follow-up including physical activity behaviour (primary outcome measure: Global Physical Activity Questionnaire), quality of life, mental wellbeing, social capital and perceptions of the built environment. Linear regression was used to calculate the mean difference between post-intervention and baseline measures adjusting for age, season, education, car ownership and deprivation. Multi-level models were fitted using a random intercept at the super output area (smallest geographical unit) to account for clustering within areas. The analyses were stratified by distance from the greenway and deprivation. We assessed change in the social patterning of outcomes over time using an ordered logit to make model-based outcome predictions across strata.

**Results:**

The mean ages of intervention samples were 50.3 (SD 18.9) years at baseline (*n* = 1037) and 51.7 (SD 19.1) years at follow-up (*n* = 968). Post-intervention, 65% (adjusted OR 0.60, 95% CI 0.35 to 1.00) of residents who lived closest to the greenway (i.e., ≤400 m) and 60% (adjusted OR, 0.64 95% CI 0.41 to 0.99) who lived furthest from the greenway (i.e.,≥1200 m) met the physical activity guidelines - 68% of the intervention sample met the physical activity guidelines before the intervention. Residents in the most deprived quintiles had a similar reduction in physical activity behaviour as residents in less deprived quintiles. Quality of life at follow-up compared to baseline declined and this decline was significantly less than in the control area (adjusted differences in mean EQ5D: -11.0 (95% CI − 14.5 to − 7.4); − 30.5 (95% CI − 37.9 to − 23.2). Significant change in mental wellbeing was not observed despite improvements in some indicators of social capital. Positive perceptions of the local environment in relation to its attractiveness, traffic and safety increased.

**Conclusions:**

Our findings illustrate the major challenge of evaluating complex urban interventions and the difficulty of capturing and measuring the network of potential variables that influence or hinder meaningful outcomes. The results indicate at this stage no intervention effect for improvements in population-level physical activity behaviour or mental wellbeing. However, they show some modest improvements for secondary outcomes including positive perceptions of the environment and social capital constructs. The public health impact of urban greenways may take a longer period of time to be realised and there is a need to improve evaluation methodology that captures the complex systems nature of urban regeneration.

**Supplementary Information:**

The online version contains supplementary material available at 10.1186/s12966-021-01213-9.

## Introduction

By 2050, 84% of Europe’s population is expected to live in cities (75% of the European population already do so) [1]. Urban ecosystems need to be improved to ensure that, above all, they are sustainable and can support a higher quality of life for growing populations. We know that the natural and built environments including urban green spaces (UGS), and their relationships to the social fabric, are critical determinants of physical and mental health, and the economic and environmental wellbeing of our cities [2–5].

However, in an increasingly urbanised world, UGS are often under threat and face extensive competition, especially from housing, business and transport demands. Improving urban health and reducing health and social inequalities can be achieved by policies and practices that effect changes to create and enhance green (e.g. parks, forests, greenways) and blue (e.g. rivers, canals, beaches) space and by creative urban design that ultimately supports populations and individuals, encouraging healthier behaviour. Various political frameworks underscore the need for UGS in our cities. For example, the New Urban Agenda [6] states that ‘green space can reduce urban poverty, including tackling urban regeneration and creating safe and social spaces for integration and interaction, and access to quality services’. Similarly, the 2030 Agenda for Sustainable Development [1] pledges to ‘provide universal access to safe, inclusive and accessible, green and public spaces, in particular, for women and children, older persons and persons with disabilities’ (SDG 11.7). Therefore, UGS can contribute to the achievement of a number of the UN’s Sustainable Development Goals.

A large body of evidence demonstrates the benefits of UGS (e.g. pocket parks, green roofs/walls, greening of vacant lots, urban trails/greenways) for better health and for mitigating inequalities [2, 3, 5, 7]. Evidence has demonstrated significant positive contributions to physical, psychological, social, economic and environmental wellbeing [8–20]. However, these benefits may not be equitably distributed across populations and some UGS have been associated with widening health and social inequalities [17]. There are plausible aetiological pathways between contact with nature and reduced risk of non-communicable diseases (NCDs) [7]. Natural environments can also provide co-benefits such as combatting air pollution, enhancing resilience to adverse weather [10], and promoting social inclusion [15]. However, some of the evidence is contested.

Much of the evidence for health benefits stems from small, short-term (quasi)experimental or cross-sectional observational studies, and to a lesser extent longitudinal observational studies, but the evidence from intervention studies is sparse [21, 22]. A previous systematic review involving 11 studies [23] suggested that there was promising evidence for UGS interventions that combined a change to the physical green space with a promotion/marketing programme (i.e. a dual approach) for increasing park usage and physical activity levels. However, that review solely focused on physical activity behaviour. A more recent study [21] extended this work to review the current evidence base of UGS interventions (involving 38 studies) for other health, social and environmental benefits to understand better the multi-functional nature and value of UGS. Hunter et al. [21] identified evidence supporting the use of certain UGS interventions for health, wellbeing (e.g., reduction in stress), social (e.g., reduction in crime, improved perceptions of safety) and environmental (e.g., increased biodiversity) benefits. Most research and policy assume that proximity and access to UGS are surrogates for use, and more UGS nearby is assumed to be good for all. However, substantial sections of the population (many with high risk of NCDs), do not visit or pay attention to UGS. Our ability to properly evaluate UGS, given the co-benefits, is limited. Researchers have yet to fully evaluate and economically value the complex, multi-functional nature of UGS and so the full potential of these spaces as public health assets has yet to be realised [21, 24]. Further, UGS tend to be viewed as discrete physical ‘assets’ without adequate appreciation of how health and other co-benefits (such as social, environmental and economic) rely on their wider integration with the surrounding urban fabric and the social environment.

The aim of this study is to investigate the public health impact of the development of an urban greenway (an UGS intervention)—the Connswater Community Greenway (CCG)—on a range of physical activity, health, wellbeing, social, and perceptions of the environment outcomes, to provide an understanding of the public health influence of a systems-level intervention. More specifically, outcomes include physical activity, general health, mental wellbeing, social capital and perceptions of environment, stratified by exposure to the CCG and deprivation.

## Methods and materials

The Physical Activity and Rejuvenation of Connswater (PARC) Study is a before-and-after evaluation of the public health effects of the CCG on physical activity, health, mental wellbeing, social and perceptions of the environment outcomes in Belfast [25]. The study was developed in partnership with statutory, voluntary and community organisations, and comprised four main elements: 1) a quasi-experimental before-and-after survey of the local CCG population (repeated cross-sectional design); 2) assessment of change in the local built environment and walkability using data from geographic information systems (GIS); 3) semi-structured interviews with local residents, and a range of community stakeholders before and after the regeneration project; 4) an economic evaluation. The current study focuses on the results from the before and after household survey. Economic evaluations have been published elsewhere [24, 26]. The study was funded by the National Prevention Research Initiative (Medical Research Council) and approved by the Office for Research Ethics Committees, Northern Ireland (09/NIR02/66).

### The intervention

#### Context

**Fig. 1:**
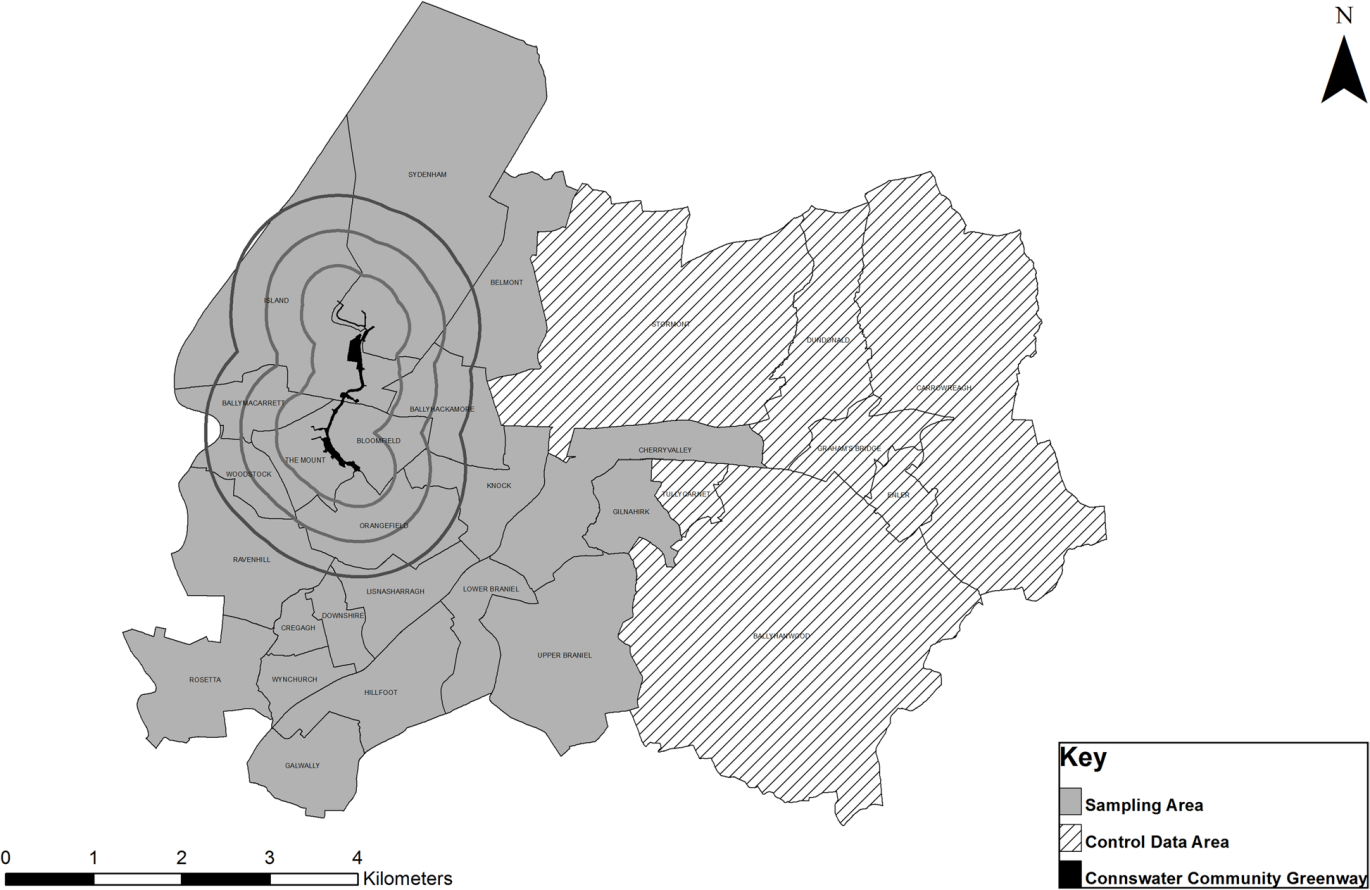
The Connswater Communitty Greenway and PARC Study Sampling Area

The study population comprised those residents living within 29 electoral wards (i.e. the smallest unit of administrative geography in Northern Ireland with an average population of 4000) in the political constituency of the CCG with a total population of approximately 110,600 (see Fig. 1). Twenty-two of these wards (approximately 87,500 residents) have a geographical centroid at or within (≤) a 1-mile radius of the CCG (i.e. the intervention area/sample), and seven of the wards are within the top 25% most deprived wards in Northern Ireland, as determined by the Northern Ireland Multiple Deprivation Measure [27].

#### Intervention description

The CCG (www.communitygreenway.co.uk) is a major urban regeneration project in Belfast, Northern Ireland, funded primarily by a Big Lottery Living Landmarks Award, and provision of other funding by local government departments and the local city council (totalling £40 m). The funding for the CCG was acquired by a local community organisation, and is jointly managed and maintained with the local authority. Specific aspects of the regeneration include: provision of a 9 km urban greenway along the course of 3 rivers; 5 km of remediated water courses; 16 km of new or improved foot and cycle paths; development of a new civic square; development of 8 tourism and heritage trails; 23 new or improved bridges or crossings; 22 new signage points; installation of public art; 13 ha of upgraded parks; 2 multi-use games areas; 2 new toilets. Many of the green spaces that became part of the CCG did exist prior to the intervention, but were mostly unconnected to each other with poor accessibility to surrounding neighbourhoods. The new paths, bridges and new access points to parks opened up these improved spaces to more of the local population. For example, one new park gate at Sir Thomas and Lady Dixon Park increased the number of households in its 5 min catchment by 59% (1702 households to 2701 households) and the 15 min catchment by 33% (12,992 households to 17,314 households). This was accompanied by extensive landscaping and enhanced biodiversity creating a wildlife corridor. Significant community engagement and involvement occurred throughout the course of the development of the CCG including in the design, provision of volunteering opportunities, and the naming of local bridges. Twenty-six schools and colleges are in close proximity to the CCG and two education officers were employed to engage the schools and colleges with the CCG. Another unique aspect was that the CCG had lighting columns along the whole route, and was lit 24 h-a-day, making it the first area of UGS to be available for use 24 h-a-day in Northern Ireland. The regeneration also involved a £11 m flood alleviation scheme, moving the course of a river away from a residential area prone to flooding. Social engagement and CCG promotion activities and events occurred in parallel with physical changes to the intervention site. A ‘bottom-up’ approach was applied which involved the employment of a full-time community support officer. This project recognised that UGS interventions are long-term investments as reflected by the 40-year management and maintenance plan for the CCG that was developed from the outset.

The original intention was for a 3-year design and build project. However, due to legal and contractual issues, several substantial delays had major implications on the research study. The original PARC Study was intended to be a 5-year study, but had to be extended by a further 3 years to accommodate these contractual delays that led to a delay in the development of the CCG.

**Fig. 2:**
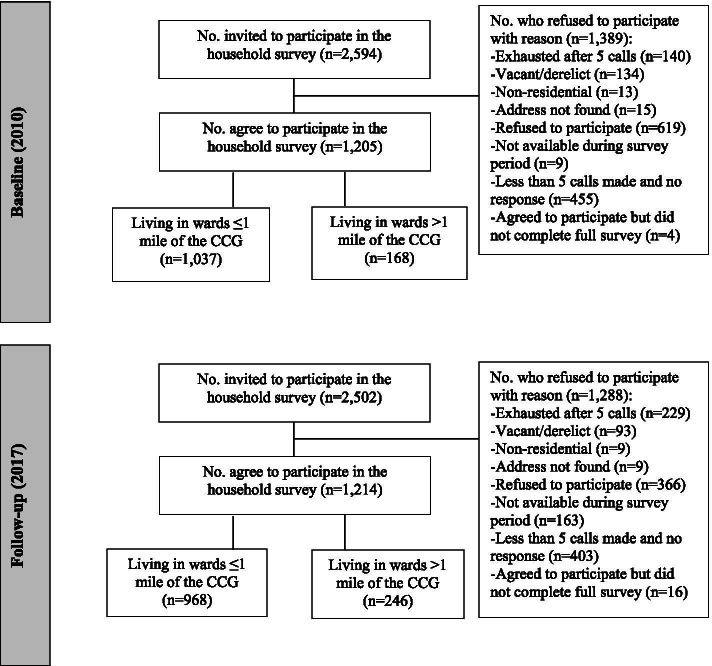
CONSORT diagram

The study design was underpinned by the socio-ecological model [28]. The survey content was informed by, and reflected the various levels of, the socio-ecological model comprising measures of individual, community and perceptions of the environment factors (Fig. 2). The aim of the CCG was to deliver positive health, mental wellbeing, social and perceptions of the environment outcomes for the local population. Primarily, the CCG was hypothesised to offer new opportunities for physical activity through the development of the linear corridor, new and improved cycle and footpaths, and promotional events and social activities to encourage usage and physical activity. In addition to the range of changes to the physical environment, other interventions to promote physical activity included neighbourhood walking groups and initiatives targeted to promote the use of the CCG in distinct population segments (e.g. young mothers, unemployed and senior citizens), schools-based initiatives and community-based social marketing initiatives. This dual approach (i.e., changes to the physical environment coupled with promotional events and programmes to encourage use) was highlighted by Hunter et al. [21, 23] as being an important component of effective UGS interventions. The environmental aesthetics of the area were improved through the provision of landscaping involving the planting of trees and shrubs, public art and remediation of water courses to improve the biodiversity of the area. Improving the perception of safety of the area was directly impacted through 24 h-a-day lighting, closed-circuit television (CCTV) and the presence of volunteer park wardens who monitor the use of the Greenway and report any problems as well as serving as community champions for the CCG.

### Study design – natural experiment

#### Repeated cross-sectional household survey

A survey of a random sample of households was conducted in 2010–2011 (i.e. before development of the CCG and repeated in 2016–2017 (i.e. 6 months after the opening of the CCG). Surveys were conducted over a 12-month period to account for seasonality. Households in 29 electoral wards were identified as the target sampling area due to their proximity to the CCG. The sampling strategy ensured proportionality with the Northern Ireland population based on estimates of the number of residents aged 16 years or older provided by the 2001 and 2011 Census. A random probability sampling framework was constructed by a random selection of addresses from each of the selected electoral wards using the Royal Mail’s Postal Address File (PAF), stratified by the proportion of the overall population within each target electoral ward. An information sheet about the study was posted to each household, followed up by a visit approximately 1–2 weeks later from a trained interviewer. The interviewer called back a maximum of five times in order to achieve a completed interview. Data collection was undertaken by an independent survey company (Perceptive Insights). If there was no response to these call backs, the address was recorded as a ‘non-response’ and another address within the same area was selected using the same process. For each household, the ‘last birthday rule’ (i.e. person in the household aged 16 years and over who had the most recent birthday) was used to randomly select an individual within each selected household to complete the survey. After the selected individual provided written consent to participate, an interviewer-administered questionnaire was completed.

#### Sample sizes

The primary outcome for the survey was the proportion of the affected population achieving the recommended levels of (total) physical activity. The sample size required to detect differences in population proportions was 934 at both time points, assuming an effect size of 0.15 at 90% power (α = 0.05), estimated using the initial population proportions of those achieving the recommended levels of physical activity using alternative assumptions of 20, 30 and 50%. We surveyed 1037 and 968 individuals, who resided in the electoral wards whose geographic centroid was ≤1-mile of the CCG area (i.e. the intervention area/sample), before and after the intervention respectively. The ≤1 mile radius represents an approximate 15-min walk to access the CCG. This distance is commonly used as a rule of thumb in walkability literature as being accessible [29–31].

In addition, we surveyed a further 168 and 246 individuals who resided in seven other wards (see Fig. 1) in the wider area (representing a similar proportion of the population to the other areas), which facilitated the exploration of exposure to the CCG via distance decay, before and after the intervention respectively. The other seven wards have a geographical centroid greater than a 1-mile radius from the CCG (i.e., the comparator area), and immediately surround the ‘intervention’ area (i.e., the wards within a 1-mile radius of the CCG).

#### Outcome measures

Physical activity was measured using the Global Physical Activity Questionnaire (GPAQ) which assessed total physical activity time via the domains of work, active travel and recreational physical activities of moderate and vigorous-intensity in the last 7 days [32]. This measure has been validated for the Northern Ireland adult population and reliably captures change in physical activity [33]. The primary outcome was the change in proportion of individuals identified as regularly physically active, according to the UK recommendations of a minimum of 150 min of at least moderate-intensity physical activity per week (or 75 min of vigorous intensity physical activity per week, or a combination of the two) [34, 35].

Secondary outcome measures included: i) mental wellbeing using the Warwick-Edinburgh Mental Wellbeing Scale [36, 37]; ii) quality of life using the EQ-5D-3L instrument [38, 39]; iii) perceptions of the characteristics of the environment associated with active travel and physical activity, including attractiveness, traffic, access to amenities and safety (measured on a 1–5 scale; 1 = strongly disagree; 5 = strongly agree) [40]; iv) items relating to neighbourhood social capital, as reflected in civic engagement, neighbourliness, social networks and support, and perceptions of the local area using the instrument employed in the UK General Household Survey [41, 42]. See Appendices A and B for ranges of the scales and further details.

#### Comparator data

There are two sources of comparator data: i) electoral wards whose geographical centroid was greater than 1 mile from the CCG (see section 2.2.2) (control area); ii) exposure to the intervention using distance decay analysis investigating outcomes related to distance from the CCG at 0-400 m, > 400 m–800 m, > 800 m-1200 m and > 1200 m (for the intervention area sample only where data was available).

Intervention exposure was defined as proximity to the CCG infrastructure, with less-exposed people living farther from the CCG acting as a comparison group for the more-exposed people living closer to the CCG. Proximity was operationalised as the distance from the participant’s home address to the nearest accessible point (e.g., accessible footpath, park entrance) to the CCG. Distance was calculated in ArcGIS 9 (Esri, Redlands, CA) using the purposefully digitised footpath network (see [43] for further details). Briefly, this included mapping the complete footpath network for the ‘intervention area’ surrounding the CCG. As no comprehensive network data had been collected on footpaths in Northern Ireland, this had to be mapped afresh, using the existing map base provided under a research agreement by the Land and Property Services of Northern Ireland cross-checked against aerial photographs, other online sources and field visits. Distances of 400 m, 800 m and 1200 m were chosen as they represent an approximate 5, 10 and 15-min walk respectively, from the participants home to the CCG. These distances have been used in previous physical activity and urban environment literature [44]. In particular, the limit of the ‘neighbourhood of opportunity’ has been described as being situated within 1000 m of home with 400 m being particularly influential [45–47].

We originally intended to compare our survey findings to a Northern Ireland population survey of physical activity (The Northern Ireland Sport and Physical Activity survey (SAPAS)) commissioned by Sport Northern Ireland [25], which also used the GPAQ [48]. This survey was collected in 2010 and was due to be repeated in 2015. However, owing to austerity in the public sector this survey was not repeated as intended. Therefore, we were unable to compare our household survey findings directly with the general Northern Ireland population as a whole. However, we have accessed other physical activity data (from a different government-funded survey), albeit using a different physical activity measurement tool (International Physical Activity Questionnaire; IPAQ [49]) and different sampling strategy, to illustrate the trend in population physical activity behaviour in adults in Northern Ireland between 2010 and 2017.

#### Statistical analyses

The main aim of the repeated cross-sectional analysis was to assess the effect of the CCG on a range of outcomes (physical activity behaviour, mental wellbeing, social environment (including social capital), perceptions of environment, exposure to the CCG). Pre–post changes in outcomes between baseline (wave 1; 2010) and follow-up (wave 2; 2017) were investigated.

For each outcome, linear regression was used to calculate the mean difference (in minutes of physical activity) at baseline (in 2010) compared with post-intervention (in 2017) (and 95% confidence interval) after adjusting for age, season, education, car ownership and area deprivation. Multi-level models were fitted using a random intercept at the super output area (individuals within super output areas) to account for clustering within areas. Analyses were repeated with physical activity category (as per groups defined by the UK government guidelines) as the outcome using logistic regression. The analyses were stratified by distance from the CCG (exposure to the intervention) and deprivation. Interaction tests were conducted by fitting interaction terms within regression models.

Where comparable outcome data were available for the region from the Health Survey for Northern Ireland (2010 and 2017), effects in the CCG intervention sample were compared with any parallel trends for the Northern Ireland adult population. Due to the different physical activity measurement instruments used for data collection, we have not undertaken any formal statistical testing but rather show trends over time (Appendix D).

To investigate effects on health inequalities, we undertook a stratified analysis to assess whether any impacts on the primary outcome were socially patterned. We assessed whether the differences in the social patterning of outcomes changed over time compared to baseline. Such analyses adopted the approach of Hunter et al. [50] using logistic regression to make model-based outcome predictions across strata. All analyses were undertaken in Stata 13 (StataCorp. Stata Statistical Software: Release 13. College Station, TX: StataCorp LP. College Station, Texas (TX): StataCorp LP; 2013).

## Results

### Population characteristics

The intervention area sample at baseline (*n* = 1037) and post-intervention (*n* = 968) had similar characteristics (Table 1). The mean ages of the intervention area samples were 50.3 (SD 18.9) years at baseline and 51.7 (SD 19.1) years at follow-up. More males were recruited at follow-up (44.5%) compared to baseline (41.0%). Samples were similar in terms of the number of participants from the most deprived quintile (22.8% at baseline versus 23.3% at follow-up).

**Table 1:** Sample Characteristics (intervention sample only (i.e. ≤1 mile radius from the greenway)

Variable	Baseline sample, ***N*** = 1037	Follow-up sample, N = 968
**Demographic**		
Male, n (%)	41.0% (425/1037)	44.5% (431/968)
Age mean (SD), years	50.3 (18.9)	51.7 (19.1)
Age group		
16–25 years	7.6% (78/1020)	7.1% (69/968)
25–35 years	18.0% (184/1020)	18.1% (175/968)
35–45 years	18.2% (186/1020)	15.7% (152/968)
45–55 years	16.5% (188/1020)	14.6% (141/968)
55–65 years	13.2% (135/1020)	16.1% (156/968)
65–75 years	13.2% (135/1020)	14.6% (134/968)
75+ years	13.1% (134/1020)	13.8% (134/968)
Marital status		
Married/Cohabiting	48.8% (506/1036)	44.9% (433/965)
Separated/Divorced/Widowed	23.4% (242/1036)	24.1% (233/965)
Single	27.8% (288/1036)	31.0% (299/965)
Number of households with children < 16 years	27.3% (283/1037)	23.2% (225/968)
Weight		
Normal or underweight	43.1% (425/985)	46.7% (435/931)
Overweight	36.8% (362/985)	37.8% (352/931)
Obese	20.1% (198/985)	15.5% (144/931)
General health		
Poor to Fair	34.3% (355/1034)	31.2% (302/968)
Good to Excellent	65.7% (679/1034)	68.8% (666/968)
Long-term illness or disability that limits daily activity	31.0% (321/1037)	33.0% (319/968)
**Socio-economic and Car/Bicycle Access**		
Educational level		
Tertiary or equivalent	34.7% (359/1036)	42.8% (414/968)
Secondary school	41.6% (431/1036)	37.4% (362/968)
None or other	23.7% (246/1036)	19.8% (192/968)
Weekly household income, £		
£60 to £230	35.6% (320/898)	24.5% (189/771)
£231 to £580	37.0% (332/898)	47.0% (362/771)
£581 or greater	27.4% (246/898)	28.5% (220/771)
Economically active^a^	52.1% (540/1037)	50.8% (492/968)
Accommodation		
Owned outright	36.4% (374/1028)	31.6% (306/968)
Mortgage/co-ownership	32.7% (336/1028)	25.9% (251/968)
Rented	30.9% (318/1028)	42.5% (411/968)
Car in household	74.5% (773/1037)	69.7% (675/968)
Adult bicycle in household	33.0% (342/1037)	31.2% (302/968)
**Geographic**		
Proximity from home to the CCG, m^b^		
< 400 m	24.8% (254/1024)	20.8% (201/966)
400 – 800 m	26.4% (270/1024)	24.4% (236/966)
> 800 m – 1200 m	22.6% (231/1024)	20.4% (197/966)
> 1200 m	26.2% (269/1024)	34.4% (332/966)
Area-level deprivation ^c^		
Most deprived (1st quintile)	22.8% (236/1037)	23.3% (226/968)
2	17.8% (185/1037)	18.7% (181/968)
3	22.7% (235/1037)	19.7% (191/968)
4	18.4% (191/1037)	21.9% (212/968)
Least deprived (5th quintile)	18.3% (190/1037)	16.3% (158/968)

^a^Defined as those in current employment at the time of the survey

^b^ Based on distance from home to nearest accessible point of the CCG using a GIS-derived footpath network

^c^ Based upon 2010 Northern Ireland Multiple Deprivation Measure

*CCG* Connswater Community Greenway, *GIS* Geographic Information Systems, *SD* Standard deviation

Figure 2 shows the participant flow diagram. For the baseline sample (2010), 2594 addresses were issued from which 1205 completed interviews were achieved (46.6%). However, where contact was made with a household, 65.7% participated (628 refused to participate or were not available during survey period). For the follow-up sample (2017), 2502 addresses were issued from which 1214 completed interviews were achieved (48.5%). However, where contact was made with a household, 69.7% participated (529 refused to participate or were not available during survey period).

### Effect of the CCG on physical activity behaviour

Table 2 presents the difference in mean primary and secondary outcomes (and proportions meeting physical activity guidelines) between baseline and follow-up for the intervention area (i.e., those living in wards ≤1 mile of the CCG). Table 3 presents the difference in mean primary and secondary outcomes (and proportions meeting physical activity guidelines) between baseline and follow-up for the intervention area versus control areas (i.e., those living in wards > 1 mile from the CCG).

**Table 2:** The difference in mean outcome between baseline and follow-up (intervention sample only)

	Baseline		Follow-up		Unadjusted^a^		Adjusted^b^	
	n	mean (SD)	n	mean (SD)	Difference in mean (95% CI)	*p*	Difference in mean (95% CI)	*p*
Proportion meeting UK physical activity guidelines % (n/N)	1037	68% (703/1037)	968	61% (592/968)	0.75 (0.62,0.90)^c^	0.002	0.65 (0.52,0.82) ^c^	< 0.001
Minutes of total physical activity per day	1037	89.9 (125.8)	968	72.6 (102.7)	−16.5 (−26.5,-6.5)	0.001	−16.0 (− 26.0,-6.0)	0.002
Minutes of physical activity excluding work per day	1037	44.2 (55.1)	968	39.8 (50.3)	−4.5 (−9.1,0.1)	0.058	−6.0 (−10.6,-1.3)	0.012
Minutes of work physical activity per day	1037	45.7 (104.2)	968	32.8 (81.7)	−12.1 (−20.3,-3.9)	0.004	−10.4 (− 18.7,-2.0)	0.015
Mental wellbeing (WEMWBS)^d^	1035	50.6 (8.7)	968	51.2 (9.4)	0.6 (− 0.2,1.3)	0.166	− 0.3 (−1.0,0.4)	0.438
Quality of life (EQ5D)^e^	1037	73.3 (20.1)	968	63.2 (30.9)	−10.0 (− 12.2,-7.8)	< 0.001	−11.1 (− 13.2,-9.0)	< 0.001
Social capital^fa,b^								
Local area trust	1032	3.4 (0.5)	968	3.5 (0.5)	0.1 (0.1,0.2)	< 0.001	0.1 (0.1,0.2)	< 0.001
Social networks	1037	4.0 (0.6)	968	3.9 (0.6)	−0.1 (− 0.2,− 0.1)	< 0.001	-0.1 (− 0.1,-0.0)	0.001
Perception of environment^g^								
Attractive	1037	3.6 (0.8)	960	3.9 (0.6)	0.3 (0.2,0.3)	< 0.001	0.3 (0.2,0.3)	< 0.001
Traffic	1037	2.7 (0.7)	946	2.9 (0.6)	0.2 (0.1,0.2)	< 0.001	0.2 (0.1,0.2)	< 0.001
Amenities	1037	3.8 (0.6)	877	3.8 (0.6)	0.0 (−0.0,0.1)	0.111	0.1 (0.0,0.1)	0.04
Safety	1037	3.5 (0.8)	921	3.7 (0.7)	0.2 (0.1,0.2)	< 0.001	0.2 (0.1,0.3)	< 0.001

*CI* Confidence intervals, *EQ5D* EuroQol 5 dimensions, *SD* Standard deviation, *WEMWBS* Warwick Edinburgh Mental Well-Being Scale

^a^Multilevel model with super output area as random intercept in unadjusted and adjusted models

^b^Adjusted for age, gender, season, education (degree, A-level, GCSE, apprenticeship, none), car ownership (none,1 or more in household), deprivation (quintiles), marital status (married, single, divorced or widowed or separated), accommodation (owner, mortgage, rented/other), limiting long term illness (yes/no), general health (fitted as a trend across 6 categories: excellent to very poor), employed(yes, no) and weekly household income (<£230, £231 to £580, >£581)

^c^Odds ratio calculated using multilevel logistic regression model with super output area as random intercept. Adjusted models contain same variables as in ^b^

^d^ Scale 14–70 with higher scores indicating greater mental wellbeing

^e^ Scale 0–100 with higher scores indicating better health

^fa^ 1 = very big problem to 4 = not a problem at all; ^fb^ 1 = never and 4 = most days

^g^ 1 = strongly disagree; 5 = strongly agree

**Table 3:** The difference in mean outcome between baseline and follow-up (intervention sample and control sample)

		Baseline		Follow-up		Unadjusted^a^		Adjusted^b^		p for interaction
		n	mean (SD)	N	mean (SD)	Difference in mean (95% CI)	*p*	Difference in mean (95% CI)	*p*	
Proportion meeting UK physical activity guidelines: % (n/total)	Int.	1037	68% (703/1037)	968	61% (592/968)	0.75 (0.62,0.90)^c^	0.002	0.65 (0.52,0.82)^c^	< 0.001	*0.119*
	Control	168	64% (108/168)	246	67% (166/246)	1.15 (0.76,1.74)^c^	0.5	1.04 (0.61,1.79)^c^	0.877	
Minutes of total physical activity per day	Int.	1037	89.9 (125.8)	968	72.6 (102.7)	−17.3 (−28.1,-6.5)	0.002	−16.8 (−29.6,-3.9)	0.012	*0.122*
	Control	168	119.1 (152.7)	246	77.9 (100.0)	−41.1 (−72.4,-9.9)	0.013	−44.4 (−82.2,-6.6)	0.024	
Minutes of total physical activity (excluding work mins) per day	Int.	1037	44.2 (55.1)	968	39.8 (50.3)	−4.4 (−9.4,0.6)	0.081	−6.1 (−11.5,-0.7)	0.028	*0.539*
	Control	168	46.2 (72.6)	246	39.8 (41.2)	−6.4 (−21.0,8.2)	0.368	−11.1 (− 25.7,3.6)	0.131	
Mental wellbeing (WEMWBS)^d^	Int.	1035	50.6 (8.7)	968	51.2 (9.4)	0.6 (−0.4,1.5)	0.226	−0.3 (−1.1,0.6)	0.524	*0.075*
	Control	168	52.2 (8.5)	246	51.3 (9.1)	−0.8 (−2.8,1.2)	0.406	−2.4 (−4.3,-0.5)	0.018	
Quality of life (EQ5D)^e^	Int.	1037	73.3 (20.1)	968	63.2 (30.9)	−10.1 (−13.9,-6.3)	< 0.001	−11.0 (− 14.5,-7.4)	< 0.001	*< 0.001*
	Control	168	76.2 (17.3)	246	54.3 (36.5)	−21.9 (−27.9,-15.9)	< 0.001	−30.5 (−37.9,-23.2)	< 0.001	
Social capital^fa,b^										
Local area trust	Int.	1032	3.4 (0.5)	968	3.5 (0.5)	0.1 (0.1,0.2)	< 0.001	0.1 (0.1,0.2)	< 0.001	*0.866*
	Control	167	3.6 (0.5)	246	3.7 (0.4)	0.2 (0.0,0.3)	0.007	0.1 (0.0,0.3)	0.012	
Social networks	Int.	1037	4.0 (0.6)	968	3.9 (0.6)	−0.1 (−0.2,−0.1)	< 0.001	−0.1 (− 0.2,-0.0)	0.003	*0.467*
	Control	165	4.0 (0.6)	246	3.9 (0.6)	−0.1 (−0.2,0.1)	0.484	−0.1 (− 0.3,0.1)	0.254	
Perception of environment^g^										
Attractive	Int.	1037	3.6 (0.8)	960	3.9 (0.6)	0.3 (0.2,0.4)	< 0.001	0.3 (0.2,0.4)	< 0.001	*0.397*
	Control	168	3.8 (0.7)	245	4.0 (0.5)	0.2 (0.0,0.4)	0.048	0.2 (−0.0,0.4)	0.074	
Traffic	Int.	1037	2.7 (0.7)	946	2.9 (0.6)	0.2 (0.1,0.2)	< 0.001	0.2 (0.1,0.2)	< 0.001	*0.438*
	Control	168	2.9 (0.8)	244	3.0 (0.6)	0.1 (−0.0,0.3)	0.14	0.1 (− 0.0,0.3)	0.064	
Amenities	Int.	1037	3.8 (0.6)	877	3.8 (0.6)	0.0 (−0.0,0.1)	0.202	0.0 (−0.0,0.1)	0.15	*0.549*
	Control	168	3.7 (0.7)	228	3.7 (0.7)	0.0 (−0.1,0.1)	0.817	-0.1 (−0.2,0.1)	0.276	
Safety	Int.	1037	3.5 (0.8)	921	3.7 (0.7)	0.2 (0.1,0.3)	0.001	0.2 (0.1,0.3)	< 0.001	*0.304*
	Control	168	3.7 (0.8)	241	3.8 (0.7)	0.1 (−0.1,0.3)	0.152	0.1 (− 0.1,0.3)	0.27	

*CI* Confidence intervals, *EQ5D* EuroQol 5 dimensions, *Int.* Intervention, *SD* Standard deviation, *WEMWBS* Warwick Edinburgh Mental Well-Being Scale

^a^Mutiple linear regression model using cluster robust standard errors with super output area as the cluster due to small numbers of participants in wards > 1 mile

^b^Adjusted for age, gender, season, education (degree, A-level, GCSE, apprenticeship, none), car ownership (none,1 or more in household), deprivation (quintiles), marital status (married, single, divorced or widowed or separated), accommodation (owner, mortgage, rented/other), limiting long term illness (yes/no), general health (fitted as a trend across 6 categories: excellent to very poor), employed(yes, no) and weekly household income (<£230, £231 to £580, >£581)

^c^Odds ratio calculated using multilevel logistic regression model with super output area as random intercept. Adjusted models contain same variables as in ^b^

^d^ Scale 14–70 with higher scores indicating greater mental wellbeing

^e^ Scale 0–100 with higher scores indicating better health

^fa^ 1 = very big problem to 4 = not a problem at all; ^fb^ 1 = never and 4 = most days

^g^ 1 = strongly disagree; 5 = strongly agree

There was a significant decline in the proportion of the local population meeting the UK physical activity guidelines. At baseline, 68% of participants met the physical guidelines, which declined to 61% at follow-up (adjusted OR 0.65; 95% CI 0.52 to 0.82; *p* = < 0.001 (Table 2)). This decline is broadly in line with the Northern Ireland population which has seen a decline of 6% of adults meeting the UK physical activity guidelines over a similar time period (see Appendix D [51];).

For the control area, 64% met physical activity guidelines at baseline versus 67% at follow-up (adjusted OR 1.04; 95% CI 0.61 to 1.79; *p* = 0.877 (Table 3), a change which was not statistically significant, nor different statistically to the change seen in the intervention area (*p*-value for interaction of 0.119).

Similarly, the mean minutes of total physical activity reduced from 89.9 min per day before the intervention to 72.6 min per day after the intervention, corresponding to a mean reduction of 16 min per day (adjusted difference in mean − 16.0, 95% CI − 26.0 to − 6.0; *p* = 0.002) (Table 2). For the control area, the mean minutes of total physical activity reduced from 119 before to 78 min per day after the intervention corresponding to a mean reduction of 44 min per day (adjusted difference in mean − 44.4, 95% CI − 82.2 to − 6.6; *p* = 0.024)), but again this is not markedly different to that seen in the intervention area (*p*-value for interaction of 0.122) (Table 3).

Table 4 presents the results of the analysis of exposure, based on distance to the CCG for the intervention sample. For the primary outcome, 64–70% of the intervention population met the physical activity guidelines before the intervention. However, post-intervention 65% (adjusted OR 0.60, 95% CI 0.35 to 1.00; *p* = 0.051) of those living closest to the CCG (i.e. ≤400 m) and 60% (adjusted OR, 0.64 95% CI 0.41 to 0.99; *p* = 0.044) of those living furthest from the CCG (i.e. ≥ 1200 m) met the physical activity guidelines.

**Table 4:** The difference in mean outcome between baseline and follow-up (intervention sample) based upon distance to the greenway

		Baseline		Follow-up		Unadjusted^a^		Adjusted^b^		p for interaction
		n	mean (SD)	N	mean (SD)	Difference in mean (95% CI)	*p*	Difference in mean (95% CI)	*p*	
Proportion meeting UK physical activity guidelines: % (n/total)	0-400 m	254	70% (178/254)	201	65% (130/201)	0.77 (0.52,1.15)^c^	0.21	0.60 (0.35,1.00)^c^	0.051	*0.676*
	400-800 m	270	64% (174/270)	236	59% (139/236)	0.82 (0.56,1.19)^c^	0.294	0.85 (0.52,1.37)^c^	0.497	
	800–1200 m	231	70% (162/231)	197	61% (121/197)	0.65 (0.43,0.99)^c^	0.043	0.51 (0.30,0.86)^c^	0.012	
	1200 m+	269	67% (181/269)	332	60% (200/332)	0.72 (0.51,1.02)^c^	0.061	0.64 (0.41,0.99)^c^	0.044	
Minutes of total physical activity per day	0-400 m	254	101.7 (148.9)	201	81.2 (104.5)	−20.5 (−44.7,3.8)	0.098	−17.2 (−39.4,5.0)	0.129	*0.739*
	400-800 m	270	91.1 (123.2)	236	74.7 (114.1)	−15.7 (−36.5,5.0)	0.138	−14.4 (−34.9,6.0)	0.167	
	800–1200 m	231	94.8 (132.2)	197	74.8 (97.5)	−24.3 (−46.4,-2.1)	0.032	− 17.8 (− 40.2,4.6)	0.119	
	1200 m+	269	75.4 (97.1)	332	64.4 (95.9)	−11.2 (−26.7,4.2)	0.154	−11.2 (−27.3,5.0)	0.176	
Minutes of total physical activity (excluding work mins) per day	0-400 m	254	47.4 (50.9)	201	37.9 (50.0)	−9.5 (−18.8,-0.2)	0.046	−10.4 (− 19.4,-1.4)	0.024	*0.403*
	400-800 m	270	41.3 (55.1)	236	38.6 (51.0)	−2.3 (−11.6,7.0)	0.63	−4.1 (−13.2,5.0)	0.378	
	800–1200 m	231	47.3 (62.1)	197	42.6 (46.1)	−4.8 (−15.3,5.7)	0.366	−6.0 (−16.6,4.6)	0.27	
	1200 m+	269	42.0 (53.2)	332	39.9 (52.4)	−2.5 (−10.9,6.0)	0.569	−2.9 (−11.6,5.8)	0.515	
Mental wellbeing (WEMWBS)^d^	0-400 m	254	50.4 (8.8)	201	52.6 (9.5)	2.2 (0.5,3.9)	0.011	0.6 (−0.9,2.1)	0.447	*0.09*
	400-800 m	270	50.1 (9.0)	236	50.3 (8.7)	0.2 (−1.3,1.8)	0.779	−0.3 (− 1.7,1.0)	0.619	
	800–1200 m	230	50.4 (9.2)	197	50.5 (10.0)	0.1 (−1.7,1.9)	0.903	0.2 (−1.3,1.8)	0.781	
	1200 m+	268	51.6 (7.9)	332	51.3 (9.2)	−0.5 (−1.9,0.8)	0.445	−0.7 (−2.0,0.5)	0.239	
Quality of life (EQ5D)^e^	0-400 m	254	71.9 (20.5)	201	63.2 (31.6)	−8.6 (−13.4,-3.8)	< 0.001	−11.4 (− 15.7,-7.1)	< 0.001	*0.910*
	400-800 m	270	72.2 (21.6)	236	61.3 (31.0)	−9.5 (−14.1,-5.0)	< 0.001	−10.7 (− 14.9,-6.6)	< 0.001	
	800–1200 m	231	74.0 (19.1)	197	61.9 (31.4)	−12.0 (− 16.9,-7.2)	< 0.001	−12.3 (− 16.8,-7.7)	< 0.001	
	1200 m+	269	75.2 (18.5)	332	65.7 (29.8)	−10.8 (− 14.7,-6.9)	< 0.001	− 10.7 (− 14.5,-6.8)	< 0.001	
Social capital^fa,b^										
Local area trust	0-400 m	254	3.4 (0.6)	201	3.5 (0.5)	0.1 (0.0,0.2)	0.004	0.1 (0.1,0.2)	0.002	
	400-800 m	268	3.4 (0.5)	236	3.5 (0.5)	0.1 (−0.0,0.1)	0.26	0.1 (−0.0,0.1)	0.215	
	800–1200 m	228	3.5 (0.6)	197	3.5 (0.6)	0.0 (−0.1,0.1)	0.701	0.1 (−0.0,0.2)	0.081	*0.164*
	1200 m+	269	3.5 (0.5)	332	3.7 (0.4)	0.2 (0.1,0.2)	< 0.001	0.2 (0.1,0.2)	< 0.001	
Social networks	0-400 m	254	4.0 (0.6)	201	3.9 (0.7)	−0.1 (−0.2,0.0)	0.058	−0.1 (− 0.2,0.0)	0.095	
	400-800 m	270	4.0 (0.6)	236	3.9 (0.7)	−0.1 (− 0.2,0.0)	0.121	− 0.0 (− 0.2,0.1)	0.422	
	800–1200 m	231	3.9 (0.6)	197	3.9 (0.6)	−0.0 (− 0.1,0.1)	0.681	− 0.0 (− 0.1,0.1)	0.95	*0.10*
	1200 m+	269	4.0 (0.5)	332	3.8 (0.7)	−0.2 (− 0.3,-0.1)	< 0.001	− 0.2 (− 0.3,− 0.1)	0.001	
Perception of environment^g^										
Attractive	0-400 m	254	3.5 (0.8)	199	3.9 (0.6)	0.3 (0.2,0.4)	< 0.001	0.3 (0.2,0.5)	< 0.001	*0.0007*
	400-800 m	270	3.4 (0.8)	233	3.8 (0.6)	0.4 (0.3,0.5)	< 0.001	0.4 (0.3,0.5)	< 0.001	
	800–1200 m	231	3.6 (0.8)	196	3.8 (0.6)	0.2 (0.1,0.4)	< 0.001	0.3 (0.2,0.5)	< 0.001	
	1200 m+	269	3.8 (0.7)	330	3.9 (0.6)	0.1 (0.0,0.2)	0.034	0.1 (−0.0,0.2)	0.132	
Traffic	0-400 m	254	2.7 (0.7)	195	2.9 (0.7)	0.2 (0.1,0.3)	0.001	0.2 (0.1,0.4)	< 0.001	*0.596*
	400-800 m	270	2.7 (0.7)	228	2.9 (0.6)	0.2 (0.1,0.3)	< 0.001	0.2 (0.1,0.3)	0.004	
	800–1200 m	231	2.8 (0.7)	195	2.9 (0.6)	0.1 (−0.0,0.2)	0.111	0.1 (0.0,0.3)	0.034	
	1200 m+	269	2.8 (0.7)	326	2.9 (0.6)	0.1 (−0.0,0.2)	0.051	0.1 (−0.0,0.2)	0.109	
Amenities	0-400 m	254	3.8 (0.6)	188	3.9 (0.6)	0.1 (−0.0,0.2)	0.176	0.1 (−0.0,0.2)	0.244	*0.0001*
	400-800 m	270	3.8 (0.6)	206	3.9 (0.6)	0.2 (0.1,0.3)	0.001	0.2 (0.1,0.3)	< 0.001	
	800–1200 m	231	3.7 (0.6)	178	3.8 (0.5)	0.0 (−0.1,0.2)	0.423	0.1 (− 0.0,0.2)	0.108	
	1200 m+	269	3.9 (0.5)	303	3.8 (0.6)	−0.1 (− 0.2,-0.0)	0.011	-0.1 (− 0.2,-0.0)	0.005	
Safety	0-400 m	254	3.4 (0.8)	192	3.6 (0.7)	0.2 (0.0,0.3)	0.018	0.2 (0.0,0.3)	0.015	*0.910*
	400-800 m	270	3.5 (0.8)	219	3.7 (0.7)	0.1 (0.0,0.3)	0.044	0.2 (0.0,0.3)	0.012	
	800–1200 m	231	3.5 (0.9)	185	3.6 (0.8)	0.1 (−0.0,0.3)	0.091	0.3 (0.1,0.4)	0.002	
	1200 m+	269	3.6 (0.8)	323	3.8 (0.7)	0.2 (0.1,0.3)	0.001	0.2 (0.1,0.3)	0.001	

*CI* Confidence intervals, *EQ5D* EuroQol 5 dimensions, *SD* Standard deviation, *WEMWBS* Warwick Edinburgh Mental Well-Being Scale

^a^Multilevel model with super output area as random intercept in unadjusted and adjusted models

^b^Adjusted for age, gender, season, education (degree, A-level, GCSE, apprenticeship, none), car ownership (none,1 or more in household), deprivation (quintiles), marital status (married, single, divorced or widowed or separated), accommodation (owner, mortgage, rented/other), limiting long term illness (yes/no), general health (fitted as a trend across 6 categories: excellent to very poor), employed(yes, no) and weekly household income (<£230, £231 to £580, >£581)

^c^Odds ratio calculated using multilevel logistic regression model with super output area as random intercept. Adjusted models contain same variables as in ^b^

^d^ Scale 14–70 with higher scores indicating greater mental wellbeing

^e^ Scale 0–100 with higher scores indicating better health

^fa^ 1 = very big problem to 4 = not a problem at all; ^fb^ 1 = never and 4 = most days

^g^ 1 = strongly disagree; 5 = strongly agree

### Effect of the CCG on mental wellbeing, quality of life, social and perceptions of the environment outcomes

There was little evidence of a difference in mean WEMWBS before (mean = 50.6) compared with after (mean = 51.2) the intervention (adjusted difference in mean = − 0.3; 95% CI − 1.1 to 0.6; *p* = 0.524) (Table 3). In the control area, there was evidence of a slight decline in mental wellbeing from before (mean = 52.2) to after (mean = 51.3) the intervention (adjusted mean difference − 2.4; 95% CI − 4.3 to − 0.5; *p* = 0.018), though this was not markedly different from the intervention area (p-value for interaction =0.075). This decline is less than the best estimates of meaningful change which have been reported as being between 3 and 8 points [52].

Table 4 shows that, for those living closest to the CCG (i.e. ≤400 m) the mean WEMWEBS at baseline was 50.4 and at follow-up was 52.6 (adjusted difference 0.6 (95% CI − 0.9 to 2.1; *p* = 0.447)) and in those living furthest from the CCG (i.e. ≤1200 m) but within the intervention sample (and intervention area) the mean WEMWEBS was 51.6 at baseline and 51.3 at follow-up (adjusted difference in mean compared with baseline of − 0.7 95% CI − 2.0 to 0.5; *p* = 0.239).

There was evidence of a decline in mean quality of life from before (EQ5D mean = 73.3) to after (mean = 63.2) the intervention (adjusted difference in mean = − 11.0; 95% CI − 14.5 to − 7.4; *p* < 0.001). In the control area, there was evidence of a greater decline in quality of life, comparing before (mean = 76.2) versus after (mean = 54.3) the intervention (adjusted mean difference − 30.5; 95% CI − 37.9 to − 23.2; *p* = < 0.001), which was markedly different from the intervention area (p for interaction = < 0.001) (Table 3). Compared with the mean before of 71.9, the mean EQ5D score in those living closest to the CCG (i.e., ≤400 m) at follow-up was 63.2 (adjusted difference − 11.4 (95% CI − 15.7 to − 7.1; *p* = < 0.001)); for those living furthest from the CCG (i.e., ≤1200 m) mean EQ5D score changed from 75.2 to 65.7 (adjusted difference − 10.7 (95% CI − 14.5 to − 6.8; *p* = < 0.001 (Table 4)).

There were mixed findings for social capital. Table 3 shows a small improvement in local area trust between baseline and follow-up (adjusted mean difference 0.1; 95% CI 0.1 to 0.2; *p* = < 0.001) and a small decline in social networks (i.e., contact with friends, family, neighbours) (adjusted mean difference − 0.1; 95% CI − 0.2 to 0.0; *p* = 0.003) for those in the intervention area. Similar trends were found for the control area (see Tables 2 and 3) but no significant interaction was found. The exposure analysis in Table 4 shows no distinct trend for social capital constructs.

Perceptions of the environment for attractiveness (adjusted mean difference 0.3; 95% CI 0.2 to 0.3; *p* = < 0.001), traffic (adjusted mean difference 0.2; 95% CI 0.1 to 0.2; *p* = < 0.001) and safety (adjusted mean difference 0.2; 95% CI 0.1 to 0.3; *p* = < 0.001) improved over time in the intervention area, but not for access to amenities (adjusted mean difference 0.1; 95% CI 0.0 to 0.1; *p* = 0.04) (Table 2). Similar trends were found for the control area (Table 3), though with no evidence for any significant interaction between the intervention and control area. The exposure analysis in Table 4 shows no distinct trend for constructs of perception of the environment in the intervention sample.

### Equity impact of the CCG

Table 5 presents the results for the primary outcome by area level deprivation to investigate the impact of the CCG on equity. Results show that those in the most deprived quintiles had a similar reduction in physical activity behaviour compared to those in the lesser deprived quintiles.

**Table 5:** Physical activity category by area deprivation and year (intervention sample)

	Actual proportion meeting target % (n/N)	OR^a^ (95% CI)	*p*	Adjusted OR^b^ (95% CI)	*p*	Predicted probability of meeting target^c^ (95% CI)
Baseline						
Deprivation						
1st quintile (most deprived)	68% (160/236)	1.00 (ref. cat.)		1.00 (ref. cat.)		82.2 (74.5,90.0)
2nd quintile	64% (118/185)	0.83 (0.53,1.31)	0.429	1.05 (0.64,1.70)	0.859	82.9 (75.6,90.2)
3rd quintile	66% (154/235)	0.92 (0.60,1.42)	0.714	0.78 (0.48,1.26)	0.306	78.3 (70.5,86.0)
4th quintile	74% (141/191)	1.34 (0.84,2.14)	0.221	1.21 (0.71,2.05)	0.481	84.9 (78.5,91.2)
5th quintile (least deprived)	68% (130/190)	1.05 (0.66,1.67)	0.835	0.95 (0.57,1.59)	0.845	81.5 (74.4,88.6)
Follow-up						
Deprivation						
1st quintile (most deprived)	59% (134/226)	1.00 (ref. cat.)		1.00 (ref. cat.)		73.3 (62.9,83.8)
2nd quintile	56% (102/181)	0.88 (0.56,1.38)	0.58	0.97 (0.60,1.58)	0.9	72.7 (62.2,83.2)
3rd quintile	63% (121/191)	1.22 (0.78,1.91)	0.386	1.04 (0.64,1.70)	0.87	74.1 (64.2,84.0)
4th quintile	67% (142/212)	1.38 (0.89,2.14)	0.156	1.36 (0.83,2.23)	0.221	78.9 (70.9,87.0)
5th quintile (least deprived)	59% (93/158)	0.99 (0.62,1.59)	0.976	1.09 (0.64,1.86)	0.755	75.0 (65.3,84.6)

*CI* Confidence intervals, *OR* Odds Ratio, *ref. cat.* reference category

^a^Odds ratios calculated from a multilevel model logistic regression with super output area as random intercept. Unadjusted model contains year and deprivation interaction and adjusted models additionally contain age, gender, season, education (degree, A-level, GCSE, apprenticeship, none), car ownership (none,1 or more in household), marital status (married, single, divorced or widowed or separated), accommodation (owner, mortgage, rented/other), limiting long term illness (yes/no), general health (fitted as a trend across 6 categories: excellent to very poor), employed(yes, no) and weekly household income (<£230, £231 to £580, >£581)

^b^P for interaction comparing OR per quintile increase in deprivation in 2010 versus 2017 after adjustment = 0.545

^c^Predicted probabilities by deprivation category calculated setting age equal to 50.3, gender to female, season to winter, education to degree, car ownership to 1 or more in household, marital status to married, accommodation to owner, limiting long term illness to none, general health to the 3rd of 6 categories from, excellent to very poor, employed to yes and income to £231 to £580

## Discussion

Our study has shown declines in physical activity behaviour in the CCG area at the follow-up period compared to baseline in the intervention area. This is broadly in line with the decrease in physical activity levels in the adult population in Northern Ireland. Results also showed a decline in quality of life at follow-up compared to baseline in the intervention area, albeit this decline was significantly less than in the control area. There was little evidence of a change in mental wellbeing in the intervention area. There was however evidence of improvements in some constructs of social capital, namely local area trust, but not for increased contact with social networks in the intervention area. Results also showed increased perceptions of the local environment for attractiveness, traffic and safety, but not for access to amenities in the intervention area. Overall, our analyses showed no evidence for a pattern of impacts for those living closer to the greenway (than those living farther away) and that these impacts were broadly similar for those living in deprived and affluent neighbourhoods.

### Compare/contrast with previous literature

Our study adds to the relatively scant literature on assessing the public health impact of urban greenways. The recent review by Hunter et al. [21] identified only six previous studies that evaluated the public health impact of urban greenways and trails. All studies that employed a dual approach (i.e., those that combined a change to the physical environment with promotion activities) demonstrated a significant intervention effect. For example, Fitzhugh et al. [53] showed significant effects for total physical activity and cycling when investigating the impact of a greenway in an urban area in Tennessee, USA. The intervention involved retrofitting 2.9 miles of urban greenway to enhance pedestrian connectivity costing $US 2.1 m. The follow-up period was 14 months after construction of the urban greenway was completed.

The IConnect study by Ogilvie et al. [54], investigated the impact of building or improving walking and cycling routes in three cities in the UK. This large quasi-experimental study (*n* = 1796 participants) conducted follow-up surveys at 1 year and 2 years. Results demonstrated that proximity to the new walking/cycling infrastructure was associated with greater usage. At 1-year follow-up, a 32% increase in usage was reported, which increased to 38% at 2-year follow-up. At 2-year follow-up, the study also showed that those living nearer the new infrastructure significantly increased walking and cycling levels (by 15 mins/week/km) and overall physical activity levels (by 12.5 mins/week/km) [55].

It is important to note that both studies [53, 55] had follow-up periods > 1 year post-construction and, in the case of Goodman et al. [55], the post-intervention effect was not realised until the 2-year follow-up period. Therefore, we could hypothesise that, given our post-intervention evaluation started only 6 months after completion of construction, it might be too early to see population-level changes in our study. Albeit, we acknowledge that our data collection took place over 12 months so some respondents could potentially have experienced the greenway for up to 18 months. The findings by Fitzhugh et al. [53] and Goodman et al. [55] suggest that it takes at least 1 year after completion of construction for the local population to become aware of the new infrastructure and to become regular users. However, these studies largely focused on physical activity behaviours and so we do not know the impact of these interventions on health, wellbeing, social or perceptions of the environment outcomes.

### Importance of context

Population level interventions do not operate in a vacuum, and so it is important to note the context in which the Connswater Community Greenway natural experiment took place [56]. The construction period and post-completion of the greenway was at a time of significant economic austerity, resulting in uncertainty regarding job security and job location; a time when health and wellbeing was at its most vulnerable. During much of this period there was also no Government Executive in place in Northern Ireland and so many policy initiatives were stalled which could have had wider effects on investment and other interventions in the area. Contextual issues of security and wider political aspects have been the subject of discussion in our previous work in this area [57].

“Natural experiments” are challenging to evaluate because this sort of added social complexity makes it difficult to control for the many factors and “moving parts” that generate community level outcomes. Population level data from the Northern Ireland Statistics and Research Agency (NISRA) [58] shows that east Belfast (i.e., the part of the city in which the greenway was developed) has worsening profiles for health and education over the 8-year period of our study. Data from other government surveys (see Appendix D) also showed a decline in levels of physical activity across the country.

Following the theory of the socio-ecological model, changes in major structural effects like land uses should drive more significant changes in behaviour (e.g., increased local destinations, more footfall, more social interaction) and land markets, particularly in a deprived area like east Belfast are very slow to evolve and stimulate change. These effects may not be discernible for many years to come. Given that the primary focus of the CCG was on connecting and improving existing greenspace, the CCG largely depends on *induced* land use change in the surrounding areas rather than the intervention itself producing such changes, as would be the case with a totally new greenway or park, or conversion of previous private land to public access.

As emphasised earlier, there was a seven-year gap between when the baseline and follow-up surveys were conducted. It is likely that societal trends during this extended period could have largely confounded the effect of the intervention. For example, we see slight change in the demography of the local population over this timeframe, such as, slightly wealthier, and more highly educated people living in rental properties.

Our findings also demonstrate that a relatively small amount of physical activity behaviour (variance/R^2^ value) was explained by our measured variables. The proportion of the variance explained was estimated to be 11% at baseline and 20% post-intervention. There are other factors affecting physical activity behaviour that we have not captured. For example, based on the socio-ecological model, genetic factors, psychological variables such as attitudes, intention to exercise, self-efficacy, physical activity history and transport environment have been identified as known correlates and determinants of adult physical activity [59]. In addition, contextual factors have been highlighted as important for the effects of built environment interventions [22].

Despite the modest improvements in some secondary outcomes, the primary outcome (proportion meeting UK physical activity guidelines) declined significantly at post-intervention follow-up. These results pose several scientific and real-world implementation challenges that are too infrequently exposed in public heath intervention trials [60], including how to balance positive and negative results when primary and secondary outcomes are discordant [61]. Some largely qualitative approaches (with interpretations contingent on programme theory) have been proposed for learning from multiple outcomes, such as Contribution Analysis with Process Tracing [62] and Ripple Effects Mapping [63], analogous to Cost Consequence analysis in health economics, while more quantitative approaches adopting a Bayesian decision framework have also been advocated [64]. There is a long-standing belief that positive results are favoured by scientific journals and that this may contribute to “publication bias”. On the other hand, some journals claim now to select articles for publication based on their contribution to the literature and welcome null results that challenge conventional wisdom or prior expectations [65]. The results from our study certainly challenged prior expectations. However, it is notoriously hard to disprove any hypothesis, and so negative studies must have the precision and strength of design to be reasonably persuasive.

### Strengths and limitations

This study took advantage of a rare natural experiment opportunity to investigate the public health impact of a new urban greenway. To the best of our knowledge, this study is one of the first to evaluate the multi-functional nature of UGS interventions, viewed through a health, wellbeing, social, perceptions of the environment and equity lens [21].

The study adds to the rather sparse evidence base investigating the impact of urban greenways which has largely been ‘opportunistic’ studies undertaking process evaluations focussing on usage, using uncontrolled pre/post study designs and collecting data using observational, intercept or e-counter methods [66]. We have also addressed a number of key limitations and sources of bias identified by Benton et al. [66] in a review detailing the risk of bias inherent in built environment interventions for promoting physical activity. For example, we published our study protocol which outlined our analytical approach a priori [25]. We also employed a number of well-matched comparator analyses including sampling survey respondents in electoral wards greater than 1 mile from the intervention site (albeit the sample size for the control group was much less that the intervention area) and investigating the impact of the greenway using distance from greenway as a measure of exposure. Since we employed a repeated, cross-sectional design to investigate population-level impacts, we had minimal missing data (as we might have anticipated with a longitudinal design). Our sampled population was also representative of both the east Belfast population and wider Northern Ireland population in terms of age, gender and deprivation profiles, variables which we adjusted for in our analyses.

Our study did have a number of limitations including reliance on the use of self-report measures in the household survey. Validated instruments were used and standardised scoring protocols employed where possible. However, it is important to note the susceptibility of self-reported measures, in particular physical activity behaviour, to social desirability and recall bias [67–69], though we do not see why this might have differed before and after the greenway construction. If this bias was greater after the construction of the greenway, this would not explain our negative findings. Our study only sampled adults aged 16 years and over, so we do not know the impact of the greenway on children or adolescents. We were unable to compare the impact of the greenway on physical activity levels of the Northern Ireland population as originally intended, as the SAPAS survey was not re-commissioned due to austerity measures. However, based on data from another population-wide Northern Ireland household survey (see Appendix D), we did report similar trends in terms of a 6% decline in adult physical activity levels across the country. As we previously highlighted, our follow-up household survey commenced approximately 6 months after completion of the greenway which may have been too short a timeframe to see population-level changes based on the findings from Fitzhugh et al. [53] and Goodman et al. [55]. However, this is similar to findings by West and Shores [70] who showed no significant change in physical activity following the construction of a new greenway. We were unable to extend this timeframe due to funding restrictions: our originally planned 5-year study became an 8-year study. Despite these limitations, our study contributes to the evidence base on UGS interventions for population health outcomes, providing data on physical, mental, social and perceptions of the environment impacts, and highlights key methodological considerations, such as the selection of suitable control samples, for the evaluation of large-scale natural experiment studies.

### Implications of findings

Our study adds to the evidence base on UGS interventions by the WHO Regional Office for Europe [3] and Hunter et al. [21] and our findings highlight the importance of viewing UGS interventions through the multi-functional lens of health, wellbeing, social, perceptions of the environment and equity impacts. To view UGS only through one lens under-evaluates the public health impact of such interventions. Our study has a particularly important message for policy-makers – such large-scale investments must be given time to mature and realise their true impact. From our qualitative data and discussions with local stakeholders, the CCG is having a significant impact on the local area in terms of increased number of visitors, attracting housing developments and business investments. The CCG is highly regarded as a vanguard project, led by the local community, stimulating interest in future greenway investments across Northern Ireland [71]. However, these effects have not, to date, been translated into the variables that were collected in this study.

### Future research

In order to address our hypothesis that the follow-up survey was conducted in too short a timeframe following completion of the greenway to realise population level changes, there is a need to conduct a further wave of the study. We also need to interrogate our longitudinal qualitative data from household interviews to further explore the reasons for our findings. This will be the subject of future publications and is beyond the scope of the current manuscript. We need new methodological approaches that can help us better understand mixed results from a range of outcomes, including those with conflicting and contrasting findings [61], enabling us to reflect on and update pre-defined logic models. Little is known about non-use of UGS, but the limited evidence suggests culture (including nature orientation), values, and capability (i.e., not wanting to or not feeling able to visit, for example, due to time poverty) are greater determinants of non-use than lack of nearby UGS which requires further exploration.

## Conclusion

Our findings showed no intervention effect for improvements in population level physical activity behaviour or health but did show some modest improvements for secondary outcomes including perceptions of environment and some constructs of social capital. Based on the results of previous similar studies, we hypothesise that the public health impact of the new urban greenway may take a longer period of time to be realised. The Connswater Community Greenway management team should continue to engage with the local communities to promote greater usage and leverage the capacity of communities for potential longer-term gains. Our findings illustrate the major challenge of evaluating complex urban interventions and the difficulty of capturing and measuring the network of potential outcomes and explanatory variables and the most appropriate time to evaluate impact.

## Supplementary Information

**Additional file 1 **: **Appendix A**. Description of the primary and secondary outcomes and measures.

**Additional file 2 **: **Appendix B**. Household Survey.

**Additional file 3 **: **Appendix C**. Control Sample Characteristics (Control area sample (i.e. >1 mile radius from the greenway).

**Additional file 4 **: **Appendix D.** Population trends in meeting physical activity recommendations in 2010 and 2016 by sex and age [Northern Ireland Health and Wellbeing Survey].

## Data Availability

The datasets used and/or analysed during the current study are available from the corresponding author on reasonable request.

## Abbreviations

aOR: Adjusted Odds Ratio
CCG: Connswater Community Greenway
CCTV: Closed-circuit Television
CI: Confidence interval
EQ-5D-3L: EuroQol-5 Dimensions-3 Levels
GIS: Geographic Information Systems
GPAQ: Global Physical Activity Questionnaire
IPAQ: International Physical Activity Questionnaire
km: Kilometre
mins: Minutes
NCD: Non-communicable disease
NISRA: Northern Ireland Statistics and Research Agency
PAF: Postal Address File
PARC: Physical Activity and the Rejuvenation of Connswater
SD: Standard deviation
SDG: Sustainable Development Goals
UGS: Urban green space
UK: United Kingdom
UN: United Nations
USA: United States of America
WEMWBS: Warwick Edinburgh Mental Wellbeing Scale
WHO: World Health Organisation
