# Supplementary material for: Investigating the physical activity, health, wellbeing, social and environmental effects of a new urban greenway: a natural experiment (the PARC study)
Source: Int J Behav Nutr Phys Act. 2021 Oct 30;18:142. doi: 10.1186/s12966-021-01213-9 (PMC8557552; doi:10.1186/s12966-021-01213-9)
Supplement: Supplementary file 1 — Additional file 1 : Appendix A. Description of the primary and secondary outcomes and measures. [file 12966_2021_1213_MOESM1_ESM.docx]

#### Appendix A: Description of the primary and secondary outcomes and measures

The following validated self-report measures were administered via a trained interviewer in person.

| **Outcomes** | **Measures** |
| --- | --- |
| *Primary outcome* | |
| **Physical Activity**  Self-report | Physical activity was assessed using the Global Physical Activity Questionnaire (GPAQ) (Bull et al, 2009; Cleland et al, 2009). GPAQ assesses levels of overall PA through the accumulation of occupational, transport, and recreational physical activities of moderate and vigorous intensity, and was developed by the World Health Organisation to allow comparison of PA across countries. Participants were asked to complete the GPAQ to provide a measure of PA.  Variables for minutes of workplace PA (minutes/week), minutes of moderate to vigorous PA (MVPA) (minutes/week) and PA levels categorised as low, moderate and high were derived using the standardised GPAQ scoring protocol  (<http://www.who.int/ncds/surveillance/steps/GPAQ%20Instrument%20and%20Analysis%20Guide%20v2.pdf>).  The primary outcome was the change in proportion of individuals identified as regularly physically active, according to the current UK recommendations of a minimum of 150 minutes of MVPA/week (Tully et al, 2013). |
| *Secondary outcomes* | |
| **Health and Wellbeing**  Quality of life and mental wellbeing | EQ-5D-3L (The EuroQol Group, 1990): This is a measure of quality of life and used to derive the health state utility measure based on five dimensions of mobility, self-care, usual activities, pain/discomfort, and anxiety/depression (0-100), and the weighted health index. The EQ-5D-5L questionnaire is based on 5 dimensions of mobility, self-care, usual activities, pain/discomfort, and anxiety/depression, and a visual analogue scale (0-100) that assesses the participants’ health state.  WEMWBS (Tennant et al, 2007; Lloyd and Devine, 2012): derived from 14 statements (with higher scores indicating better mental health). The WEMWBS comprises 14 positively worded statements, where scores are summed with higher scores indicating greater mental well-being. |
| **Built Environment**  Perception of environment | Perception of environment (Ogilvie et al, 2008):  Attractive *(average of 3 items: F10 a, h, n) e.g. pleasant to walk: 1 strongly disagree - 5 strongly agree*  Traffic *(average of 4 items: F10 b, d, k, m) e.g. little traffic noise:1 strongly disagree - 5 strongly agree*  Amenities *(average of 5 items: F10 c, e, g, j, l) e.g. park within walking distance: 1 strongly disagree - 5 strongly agree*  Safety *(average of 2 items: F10 f,i) e.g. safe to walk after dark 1 strongly disagree - 5 strongly agree* |
| Social environment | Local area trust *(average of 8 items: G2-9) e.g. problem people being drunk: 1 very big problem to 4 not a problem at all*  Social networks *(average of 5 items: G11-15) e.g. how often contact friends: 1 never – 5 most days (scale reversed)* |
| Socio-demographic factors | Age (mean, SD); sex (% male); marital status (married/cohabiting; separated/divorced/widowed; single); number of households with children < 16 years old; educational level (tertiary or equivalent; secondary school; none or other); weekly household income (£60-£230; £231-£580; £581 or greater; economically inactive); accommodation (owned outright; mortgage/co-ownership; rented); car in household; adult bicycle in household; weight status (normal or underweight; overweight; obese) based on self-report height and weight to calculate BMI; general health (poor-fair; good-excellent), long-term illness |
|  |  |

BMI: Body Mass Index; EQ-5D: Euroqol 5 dimensions; GPAQ: Global Physical Activity Questionnaire; MVPA: Minutes of moderate to vigorous physical activity; PA: Physical activity; WEMWBS: Warwick-Edinburgh Mental Wellbeing Scale

**References:**

Bull FC, Maslin TS, Armstrong T. Global physical activity questionnaire (GPAQ): nine country reliability and validity study. J Phys Act Health. 2009;6(6):790-804. doi: 10.1123/jpah.6.6.790.

Cleland CL, Hunter RF, Kee F, Cupples ME, Sallis JF, Tully MA. Validity of the global physical activity questionnaire (GPAQ) in assessing levels and change in moderate-vigorous physical activity and sedentary behaviour. BMC Public Health. 2014;14:1255. doi: 10.1186/1471-2458-14-1255.

Lloyd K, Devine P. Psychometric properties of the Warwick–Edinburgh Mental Well-being Scale (WEMWBS) in Northern Ireland. J Ment Heal. 2012;21(3):257-63. doi: 10.3109/09638237.2012.670883.

Ogilvie D, Mitchell R, Mutrie N, Petticrew M, Platt S. Perceived characteristics of the environment associated with active travel: development and testing of a new scale. Int J Behav Nutr Phys Act. 2008;5:32. doi: 10.1186/1479-5868-5-32.

Tennant R, Hiller L, Fishwick R, et al. The Warwick-Edinburgh Mental Well-being Scale (WEMWBS): development and UK validation. Health Qual. Life Outcomes 2007;5(1):63. doi: 10.1186/1477-7525-5-63.

The EuroQol Group. EuroQol: a new facility for the measurement of health-related quality of life. Health Policy. 1990;16:199-208. doi: 10.1016/0168-8510(90)90421-9.

Tully MA, Hunter RF, McAneney H, Cupples ME, Donnelly M, Ellis G, Hutchinson G, Prior L, Stevenson M, Kee F. Physical activity and the rejuvenation of Connswater (PARC study): protocol for a natural experiment investigating the impact of urban regeneration on public health. BMC Public Health. 2013;13:774. doi: 10.1186/1471-2458-13-774.
