# Supplementary material for: Investigating the physical activity, health, wellbeing, social and environmental effects of a new urban greenway: a natural experiment (the PARC study)
Source: Int J Behav Nutr Phys Act. 2021 Oct 30;18:142. doi: 10.1186/s12966-021-01213-9 (PMC8557552; doi:10.1186/s12966-021-01213-9)
Supplement: Supplementary file 2 — Additional file 2 : Appendix B. Household Survey. [file 12966_2021_1213_MOESM2_ESM.docx]

Appendix B: Household Survey

Section A:

| **A1** | **Record gender** |  |  |
| --- | --- | --- | --- |
|  | Male | 1 |  |
|  | Female | 2 |  |
|  | Other | 3 |  |

**Respondents to be aged 16 or older**

| **A2** | What was your age last birthday? | |  |  |
| --- | --- | --- | --- | --- |
|  | **RECORD EXACT AGE** |  |  |  |
|  | 16 to 24 | | 1 |  |
|  | 25 to 34 | | 2 |  |
|  | 35 to 44 | | 3 |  |
|  | 45 to 54 | | 4 |  |
|  | 55 to 64 | | 5 |  |
|  | 65 to 74 | | 6 |  |
|  | 75 plus | | 7 |  |
|  | Refused | | 8 |  |
|  |  | |  |  |
|  | Estimate | | 1 |  |

| **A3** | What is your marital status? |  |  |
| --- | --- | --- | --- |
|  | Married (spouse in household | 1 |  |
|  | Married (spouse not living in household) | 2 |  |
|  | Cohabiting | 3 |  |
|  | Single (never married) | 4 |  |
|  | Separated | 5 |  |
|  | Divorced | 6 |  |
|  | Widowed | 7 |  |
|  | Refused | 8 |  |

| **A4** | Is your accommodation: |  |  |
| --- | --- | --- | --- |
|  | Owned outright | 1 |  |
|  | Being bought with mortgage/loan | 2 |  |
|  | Co-ownership | 3 |  |
|  | Rented from NI Housing Executive | 4 |  |
|  | Rented from housing association | 5 |  |
|  | Rented privately | 6 |  |
|  | Rented from employer | 7 |  |
|  | Rent-free | 8 |  |
|  | Squat | 9 |  |
|  | Other – specify | 10 |  |
|  |  |  |  |

Section B:

Next I am going to ask you about the time you spend doing different types of physical activity in a typical week. Please answer these questions even if you do not consider yourself to be a physically active person.

**Activity at work**

Think first about the time you spend doing work. Think of **work** as the things that you have to do such as paid or unpaid work, study/training, household chores, harvesting food/crops, fishing or hunting for food, seeking employment.

In answering the following questions:

**'vigorous-intensity activities'** are activities that require hard physical effort and cause large increases in breathing or heart rate;

**'moderate-intensity activities'** are activities that require moderate physical effort and cause small increases in breathing or heart rate.

| **B1** | Does your work involve **vigorous**-intensity activity that causes large increases in breathing or heart rate like *[carrying or lifting heavy loads, digging or construction work*] for at least 10 minutes continuously? | | |
| --- | --- | --- | --- |
|  | Yes | 1 |  |
|  | No | 2 | **Go to B4** |

| **B2** | In a typical week, on how many days do you do **vigorous** intensity activities as part of your work? | | |
| --- | --- | --- | --- |
|  | One | 1 |  |
|  | Two | 2 |  |
|  | Three | 3 |  |
|  | Four | 4 |  |
|  | Five | 5 |  |
|  | Six | 6 |  |
|  | Seven | 7 |  |
|  | None | 8 |  |

| **B3** | How much time do you spend doing **vigorous**-intensity activities at work on a typical day? | | | |
| --- | --- | --- | --- | --- |
|  |  | Hours | Mins |  |
|  |  |  |  |  |

| **B4** | Does your work involve **moderate**-intensity activity that causes small increases in breathing or heart rate such as brisk walking [or carrying light loads] for at least 10 minutes continuously? | | |
| --- | --- | --- | --- |
|  | Yes | 1 |  |
|  | No | 2 | **Go to B7** |

| **B5** | In a typical week, on how many days do you do **moderate** intensity activities as part of your work? | | | |
| --- | --- | --- | --- | --- |
|  | One | | 1 |  |
|  | Two | | 2 |  |
|  | Three | | 3 |  |
|  | Four | | 4 |  |
|  | Five | | 5 |  |
|  | Six | | 6 |  |
|  | Seven | | 7 |  |
|  | None | | 8 |  |
| **B6** | How much time do you spend doing **moderate**-intensity activities at work on a typical day? | | | |
|  |  | Hours | Mins |  |
|  |  |  |  |  |

**Travel to and from places**

The next questions exclude the physical activities at work that you have already mentioned.

Now I would like to ask you about the usual way you travel to and from places. For example to work, for shopping, to market, to place of worship.

| **B7** | Do you walk or use a bicycle (pedal cycle) for at least 10 minutes continuously to get to and from places? | | |
| --- | --- | --- | --- |
|  | Yes | 1 |  |
|  | No | 2 | **Go to B10** |

| **B8** | In a typical week, on how many days do you walk or bicycle for at least 10 minutes continuously to get to and from places? | | |
| --- | --- | --- | --- |
|  | One | 1 |  |
|  | Two | 2 |  |
|  | Three | 3 |  |
|  | Four | 4 |  |
|  | Five | 5 |  |
|  | Six | 6 |  |
|  | Seven | 7 |  |
|  | None | 8 |  |

| **B9** | How much time do you spend walking or bicycling for travel on a typical day? | | | |
| --- | --- | --- | --- | --- |
|  |  | Hours | Mins |  |
|  |  |  |  |  |

**Recreational activities**

The next questions exclude the work and transport activities that you have already mentioned.

Now I would like to ask you about sports, fitness and recreational activities (leisure).

| **B10** | Do you do any **vigorous**-intensity sports, fitness or recreational (leisure) activities that cause large increases in breathing or heart rate like running or football, for at least 10 minutes continuously? | | |
| --- | --- | --- | --- |
|  | Yes | 1 |  |
|  | No | 2 | **Go to B13** |

| **B11** | In a typical week, on how many days do you do **vigorous** intensity sports, fitness or recreational (*leisure*) activities? | | |
| --- | --- | --- | --- |
|  | One | 1 |  |
|  | Two | 2 |  |
|  | Three | 3 |  |
|  | Four | 4 |  |
|  | Five | 5 |  |
|  | Six | 6 |  |
|  | Seven | 7 |  |
|  | None | 8 |  |

| **B12** | How much time do you spend doing **vigorous**-intensity sports, fitness or recreational activities on a typical day? | | | |
| --- | --- | --- | --- | --- |
|  |  | Hours | Mins |  |
|  |  |  |  |  |

| **B13** | Do you do any **moderate**-intensity sports, fitness or recreational *(leisure*) activities that causes a small increase in breathing or heart rate such as brisk walking*,* cycling, swimming, volleyball for at least 10 minutes continuously? | | |
| --- | --- | --- | --- |
|  | Yes | 1 |  |
|  | No | 2 | **Go to B16** |

| **B14** | In a typical week, on how many days do you do **moderate**-intensity sports, fitness or recreational (*leisure*) activities? | | |
| --- | --- | --- | --- |
|  | One | 1 |  |
|  | Two | 2 |  |
|  | Three | 3 |  |
|  | Four | 4 |  |
|  | Five | 5 |  |
|  | Six | 6 |  |
|  | Seven | 7 |  |
|  | None | 8 |  |

| **B15** | How much time do you spend doing **moderate**-intensity sports, fitness or recreational (*leisure*) activities on a typical day? | | | |
| --- | --- | --- | --- | --- |
|  |  | Hours | Mins |  |
|  |  |  |  |  |

**Sedentary behaviour**

The following question is about sitting or reclining at work, at home, getting to and from places, or with friends including time spent sitting at a desk, sitting with friends, travelling in car, bus, train, reading, eating, playing cards or watching television, but do not include time spent sleeping.

| **B16** | How much time do you usually spend sitting or reclining on a typical day? | | | |
| --- | --- | --- | --- | --- |
|  |  | Hours | Mins |  |
|  |  |  |  |  |

Section C: Feelings and thoughts

| **C1** | | Below are some statements about feelings and thoughts.  Please circle the number that best describes your experience of each over the  **last** **2 weeks** | | | | | |
| --- | --- | --- | --- | --- | --- | --- | --- |
|  | |  | **None of the time** | **Rarely** | **Some of the time** | **Often** | **All of the time** |
|  | I’ve been feeling optimistic about the future | |  |  |  |  |  |
|  |  |  | 1 | 2 | 3 | 4 | 5 |
|  |  |  |  |  |  |  |  |
|  | I’ve been feeling useful | |  |  |  |  |  |
|  |  |  | 1 | 2 | 3 | 4 | 5 |
|  |  |  |  |  |  |  |  |
|  | I’ve been feeling relaxed | |  |  |  |  |  |
|  |  |  | 1 | 2 | 3 | 4 | 5 |
|  |  |  |  |  |  |  |  |
|  | I’ve been feeling interested in other people | |  |  |  |  |  |
|  |  |  | 1 | 2 | 3 | 4 | 5 |
|  |  |  |  |  |  |  |  |
|  | I’ve had energy to spare | |  |  |  |  |  |
|  |  |  | 1 | 2 | 3 | 4 | 5 |
|  |  |  |  |  |  |  |  |
|  | I’ve been dealing with problems well | |  |  |  |  |  |
|  |  |  | 1 | 2 | 3 | 4 | 5 |
|  |  |  |  |  |  |  |  |
|  | I’ve been thinking clearly | |  |  |  |  |  |
|  |  |  | 1 | 2 | 3 | 4 | 5 |
|  |  |  |  |  |  |  |  |
|  | I’ve been feeling good about myself | |  |  |  |  |  |
|  |  |  | 1 | 2 | 3 | 4 | 5 |
|  |  |  |  |  |  |  |  |
|  | I’ve been feeling close to other people | |  |  |  |  |  |
|  |  |  | 1 | 2 | 3 | 4 | 5 |
|  |  |  |  |  |  |  |  |
|  | I’ve been feeling confident | |  |  |  |  |  |
|  |  |  | 1 | 2 | 3 | 4 | 5 |
|  |  |  |  |  |  |  |  |
|  | I’ve been able to make up my mind about things | |  |  |  |  |  |
|  |  |  | 1 | 2 | 3 | 4 | 5 |
|  |  |  |  |  |  |  |  |
|  | I’ve been feeling loved | |  |  |  |  |  |
|  |  |  | 1 | 2 | 3 | 4 | 5 |
|  |  |  |  |  |  |  |  |
|  | I’ve been interested in new things | |  |  |  |  |  |
|  |  |  | 1 | 2 | 3 | 4 | 5 |
|  |  |  |  |  |  |  |  |
|  | I’ve been feeling cheerful | |  |  |  |  |  |
|  |  |  | 1 | 2 | 3 | 4 | 5 |
|  |  |  |  |  |  |  |  |

Section D: Health

This survey asks for your views about your health. This information will help you keep track of how you feel and how well you are able to do your usual activities.
Answer every question by selecting the answer as indicated. If you are unsure about how to answer a question, please give the best answer you can.
For each of the following questions, please mark an [x] in the one box that best describes your answer.

**D1** Overall, how would you rate your health during the **past four weeks**?

|  | Excellent | Very  Good | Good | Fair | Poor | Very poor |  |
| --- | --- | --- | --- | --- | --- | --- | --- |
|  | 1 | 2 | 3 | 4 | 5 | 6 |  |

**D2** During the PAST FOUR WEEKS, how much did physical health problems limit your

usual physical activities (such as walking or climbing stairs)?

|  | Not at all | Very little | Somewhat | Quite a lot | Could not do daily activities |  |
| --- | --- | --- | --- | --- | --- | --- |
|  | 1 | 2 | 3 | 4 | 5 |  |

**D3** During the PAST FOUR WEEKS, how much difficulty did you have doing your daily

work, both at home and away from home, because of your physical health?

|  | Not at all | Very little | Somewhat | Quite a lot | Could not do daily work |  |
| --- | --- | --- | --- | --- | --- | --- |
|  | 1 | 2 | 3 | 4 | 5 |  |

**D4** How much BODILY pain have you had during the past FOUR WEEKS?

|  | None | Very mild | Mild | Moderate | Severe | Very severe |  |
| --- | --- | --- | --- | --- | --- | --- | --- |
|  | 1 | 2 | 3 | 4 | 5 | 6 |  |

**D5** During the PAST FOUR WEEKS, how much energy did you have?

|  | Very much | Quite a lot | Some | A little | None |  |
| --- | --- | --- | --- | --- | --- | --- |
|  | 1 | 2 | 3 | 4 | 5 |  |

**D6** During the PAST FOUR WEEKS, how much did your physical health or emotional

problems limit your usual social activities with family or friends?

|  | Not at all | Very little | Somewhat | Quite a lot | Could not do social activities |  |
| --- | --- | --- | --- | --- | --- | --- |
|  | 1 | 2 | 3 | 4 | 5 |  |

**D7** During the PAST FOUR WEEKS, how much have your been bothered by emotional

problems (such as felling anxious, depressed or irritable)?

|  | Not at all | Slightly | Moderately | Quite a lot | Extremely |  |
| --- | --- | --- | --- | --- | --- | --- |
|  | 1 | 2 | 3 | 4 | 5 |  |

**D8** During the PAST FOUR WEEKS, how much did personal or emotional problems

keep you from doing your usual work, school or other daily activities?

|  | Not at all | Very little | Somewhat | Quite a lot | Could not do daily activities |  |
| --- | --- | --- | --- | --- | --- | --- |
|  | 1 | 2 | 3 | 4 | 5 |  |

Section E: Health today

By circling a number in one box in each group below, please indicate which statements best describe your own health state **today.**

| **E1** | **Mobility:** |  |  |
| --- | --- | --- | --- |
|  | I have no problems in walking about | 1 |  |
|  | I have some problems in walking about | 2 |  |
|  | I am confined to bed | 3 |  |

| **E2** | **Self-care:** |  |  |
| --- | --- | --- | --- |
|  | I have no problems with self-care | 1 |  |
|  | I have some problems washing or dressing myself | 2 |  |
|  | I am unable to wash or dress myself | 3 |  |

| **E3** | **Usual Activities** (e.g. work, study, housework, family or  leisure activities) |  |  |
| --- | --- | --- | --- |
|  | I have no problems with performing my usual activities | 1 |  |
|  | I have some problems with performing my usual activities | 2 |  |
|  | I am unable to perform my usual activities | 3 |  |

| **E4** | **Pain/Discomfort** |  |  |
| --- | --- | --- | --- |
|  | I have no pain or discomfort | 1 |  |
|  | I have moderate pain or discomfort | 2 |  |
|  | I have extreme pain or discomfort | 3 |  |

| **E5** | **Anxiety/Depression** |  |  |
| --- | --- | --- | --- |
|  | I am not anxious or depressed | 1 |  |
|  | I am moderately anxious or depressed | 2 |  |
|  | I am extremely anxious or depressed | 3 |  |

**E6** To help people say how good or bad a health state is, we have drawn a scale (rather like a thermometer) on which the best state you can imagine is marked 100 and the worst state you can imagine is marked 0.

We would like you to indicate on this scale how good or bad your own health is today, in your opinion. Please do this by **drawing a line from the box** below to whichever point on the scale indicates how good or bad your health state is **today**.

| **Best imaginable health state**  Example | | 100 |  |  |
| --- | --- | --- | --- | --- |
|  |  |  |  |  |
|  |  |  |  |  |
|  | |  |  |  |
|  | |  |  |  |
| 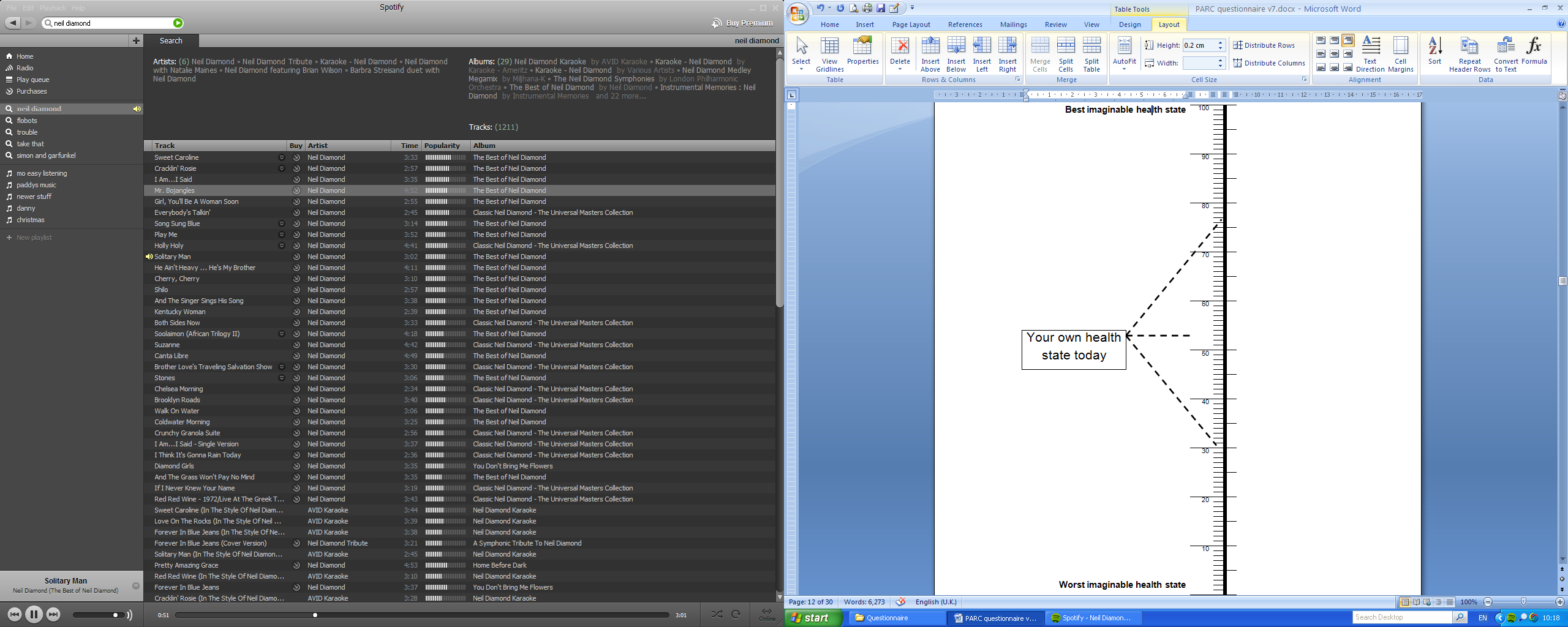 | |  |  |  |
|  | |  |  |  |
|  | |  |  |  |
|  | |  |  |  |
|  | |  |  |  |
|  | | 90 |  |  |
|  | |  |  |  |
|  | |  |  |  |
|  | |  |  |  |
|  | |  |  |  |
|  | |  |  |  |
|  | |  |  |  |
|  | |  |  |  |
|  | |  |  |  |
|  | |  |  |  |
|  | | 80 |  |  |
|  | |  |  |  |
|  | |  |  |  |
|  | |  |  |  |
|  | |  |  |  |
|  | |  |  |  |
|  | |  |  |  |
|  | |  |  |  |
|  | |  |  |  |
|  | |  |  |  |
|  | | 70 |  |  |
|  | |  |  |  |
|  | |  |  |  |
|  | |  |  |  |
|  | |  |  |  |
|  | |  |  |  |
|  | |  |  |  |
|  | |  |  |  |
|  | |  |  |  |
|  | |  |  |  |
|  | | 60 |  |  |
|  | |  |  |  |
|  | |  |  |  |
|  | |  |  |  |
|  | |  |  |  |
|  | |  |  |  |
| Your own health state today |  |  |  |  |
|  |  |  |  |  |
|  |  |  |  |  |
|  |  |  |  |  |
|  |  | 50 |  |  |
|  |  |  |  |  |
|  |  |  |  |  |
|  |  |  |  |  |
|  | |  |  |  |
|  | |  |  |  |
|  | |  |  |  |
|  | |  |  |  |
|  | |  |  |  |
|  | |  |  |  |
|  | | 40 |  |  |
|  | |  |  |  |
|  | |  |  |  |
|  | |  |  |  |
|  | |  |  |  |
|  | |  |  |  |
|  | |  |  |  |
|  | |  |  |  |
|  | |  |  |  |
|  | |  |  |  |
|  | | 30 |  |  |
|  | |  |  |  |
|  | |  |  |  |
|  | |  |  |  |
|  | |  |  |  |
|  | |  |  |  |
|  | |  |  |  |
|  | |  |  |  |
|  | |  |  |  |
|  | |  |  |  |
|  | | 20 |  |  |
|  | |  |  |  |
|  | |  |  |  |
|  | |  |  |  |
|  | |  |  |  |
|  | |  |  |  |
|  | |  |  |  |
|  | |  |  |  |
|  | |  |  |  |
|  | |  |  |  |
|  | | 10 |  |  |
|  | |  |  |  |
|  | |  |  |  |
|  | |  |  |  |
|  | |  |  |  |
|  | |  |  |  |
|  | |  |  |  |
| **Worst imaginable health state** | |  |  |  |
|  |  |  |  |  |
|  |  |  |  |  |

Section F: Neighbourhood Walkability

The next set of questions are about what it is like to walk around your neighbourhood.

**About you and your household**

| **F1** | Do you have access to a bicycle? | | |
| --- | --- | --- | --- |
|  | Yes | 1 |  |
|  | No | 2 |  |

| **F2** | How many cars or vans are owned, or available for use, by members of your household?  *Do not include motorcycles, scooters or mopeds.* | | | |
| --- | --- | --- | --- | --- |
|  |  |  |  |  |

| **F3** | How many other people live in your household?  *We mean people who have your accommodation as their only or main residence, and who either share at least one meal a day with you or share the living accommodation (living room or sitting room) with you.* | | | |
| --- | --- | --- | --- | --- |
|  |  |  |  |  |
|  | Adults aged 16 and over  **(do not include yourself)** |  |  |  |
|  | Children aged under 5 |  |  |  |
|  | Children aged between 5 and 15 |  |  |  |

| **F4** | Thinking about the work you do, which of these best describes your situation at present?  *Please answer for both yourself and your partner if applicable* | | |
| --- | --- | --- | --- |
|  |  | **You** | **Your partner** |
|  | Doing paid work full time | 1 | 1 |
|  | Doing paid work part time | 2 | 2 |
|  | Retired | 3 | 3 |
|  | Full time student | 4 | 4 |
|  | Unemployed | 5 | 5 |
|  | Disabled, invalid or permanently sick | 6 | 6 |
|  | Caring for home and family or dependants | 7 | 7 |
|  | Other | 8 | 8 |
|  | Not living with a spouse or partner |  | 9 |

| **F5** | Do you have any long-term illness, health problem or disability which limits your daily activities or the work you can do? Include problems which are due to old age. | | |
| --- | --- | --- | --- |
|  | Yes | 1 |  |
|  | No | 2 |  |

| **F6** | How tall are you? (with your shoes off) | | | |
| --- | --- | --- | --- | --- |
|  |  | Feet | Inches |  |
|  |  |  |  |  |
|  | (or | cms) |  |  |
|  |  |  |  |  |

| **F7** | How much do you weigh? (in light indoor clothes) | | | |
| --- | --- | --- | --- | --- |
|  |  | Stone | lbs |  |
|  |  |  |  |  |
|  | (or | kgs) |  |  |
|  |  |  |  |  |

**About your local area**

This section asks for your views about your **local area**. Think of your local area as everywhere within a ten-minute walk (about half a mile) from your home in East Belfast.

| **F8** | How long have you lived in your local area?  *If you have lived this area previously and come back again, please just answer about the current period of time that you have lived in your local area.* | | | |
| --- | --- | --- | --- | --- |
|  |  | Years | Months |  |
|  |  |  |  |  |

| **F9** | Looking at the faces scale, which face shows best how you feel about living in your local area? (*circle the appropriate number*). |
| --- | --- |
|  |  |

| **F10** | For each of the following statements about your local area, how strongly you agree or disagree | | | | | |  |
| --- | --- | --- | --- | --- | --- | --- | --- |
|  | **In my local area…** | Strongly agree | Agree | Neither nor | Disagree | Strongly disagree | Don’t know |
| a | It is pleasant to walk | 1 | 2 | 3 | 4 | 5 | 6 |
|  |  |  |  |  |  |  |  |
| b | There is a lot of traffic noise | 1 | 2 | 3 | 4 | 5 | 6 |
|  |  |  |  |  |  |  |  |
| c | There is a park within walking distance | 1 | 2 | 3 | 4 | 5 | 6 |
|  |  |  |  |  |  |  |  |
| d | The roads are dangerous for cyclists | 1 | 2 | 3 | 4 | 5 | 6 |
|  |  |  |  |  |  |  |  |
| e | There is convenient public transport | 1 | 2 | 3 | 4 | 5 | 6 |
|  |  |  |  |  |  |  |  |
| f | People are likely to be attacked | 1 | 2 | 3 | 4 | 5 | 6 |
|  |  |  |  |  |  |  |  |
| g | There are convenient routes for cycling | 1 | 2 | 3 | 4 | 5 | 6 |
|  |  |  |  |  |  |  |  |
| h | There is little green space | 1 | 2 | 3 | 4 | 5 | 6 |
|  |  |  |  |  |  |  |  |
| i | It is safe to walk after dark | 1 | 2 | 3 | 4 | 5 | 6 |
|  |  |  |  |  |  |  |  |
| j | The nearest shops are too far to walk to | 1 | 2 | 3 | 4 | 5 | 6 |
|  |  |  |  |  |  |  |  |
| k | There is little traffic | 1 | 2 | 3 | 4 | 5 | 6 |
|  |  |  |  |  |  |  |  |
| l | There are no convenient routes for walking | 1 | 2 | 3 | 4 | 5 | 6 |
|  |  |  |  |  |  |  |  |
| m | It is safe to cross the road | 1 | 2 | 3 | 4 | 5 | 6 |
|  |  |  |  |  |  |  |  |
| n | The surroundings are unattractive | 1 | 2 | 3 | 4 | 5 | 6 |

Section G: Social capital

Now I would like to ask you a few questions about your **immediate neighbourhood**, by which I mean **your street or block**.

| **G1** | How much of a problem are each of the following… | | | | | |  |
| --- | --- | --- | --- | --- | --- | --- | --- |
|  | Please use the following scale | Very big problem | | | | | 1 |
|  |  | Fairly big problem | | | | | 2 |
|  |  | Not a very big problem | | | | | 3 |
|  |  | Not a problem at all | | | | | 4 |
|  |  | It’s not a problem but it happens | | | | | 5 |
|  |  | |  |  |  |  |  |
|  |  | | Very big | Fairly big | Not very big | Not at all | It happens |
| **G2** | People being drunk or rowdy in public places? | | 1 | 2 | 3 | 4 | 5 |
| **G3** | Rubbish or litter lying around? | | 1 | 2 | 3 | 4 | 5 |
| **G4** | Vandalism, graffiti and other deliberate damage to property or vehicles? | | 1 | 2 | 3 | 4 | 5 |
| **G5** | People using or dealing drugs? | | 1 | 2 | 3 | 4 | 5 |
| **G6** | People being attacked or harassed because of their religion (Catholic or Protestant)? | | 1 | 2 | 3 | 4 | 5 |
| **G7** | People being attacked or harassed because of their skin colour, ethnic origin? | | 1 | 2 | 3 | 4 | 5 |
| **G8** | Teenagers hanging around on the street? | | 1 | 2 | 3 | 4 | 5 |
| **G9** | Problem with troublesome neighbours? | | 1 | 2 | 3 | 4 | 5 |

The next questions are about how often you personally contact relatives, friends and neighbours

| **G10** | How often do you do each of the following: | | | | | |  |
| --- | --- | --- | --- | --- | --- | --- | --- |
|  | Please use the following scale | On most days | | | | | 1 |
|  |  | Once or twice a week | | | | | 2 |
|  |  | Once or twice a month | | | | | 3 |
|  |  | Less often than once a month | | | | | 4 |
|  |  | Never | | | | | 5 |
|  |  | |  |  |  |  |  |
|  |  | | **Most days** | **1-2 a week** | **1-2 a month** | **Less often** | **Never** |
| **G11** | Speak to relatives on the phone | | 1 | 2 | 3 | 4 | 5 |
| **G12** | Speak to friends on the phone | | 1 | 2 | 3 | 4 | 5 |
| **G13** | Speak to neighbours (face to face) | | 1 | 2 | 3 | 4 | 5 |
| **G14** | Meet with relatives who are not living with you | | 1 | 2 | 3 | 4 | 5 |
| **G15** | Meet up with friends | | 1 | 2 | 3 | 4 | 5 |

Section H: Education and lifestyle characteristics

| **H1** | Are you still at school? |  |  |
| --- | --- | --- | --- |
|  | Still at school | 1 | **Go to H5** |
|  | Left school | 2 |  |
|  | Never went to school | 3 |  |

| **H2** | How old were you when you left school (ie elementary, secondary or grammar)? | | | |
| --- | --- | --- | --- | --- |
|  |  |  |  |  |

| **H3** | How old were you when you left full-time continuous education? | | | |
| --- | --- | --- | --- | --- |
|  |  |  |  |  |

| **H4** | How old were you when you finished your last (full- or part time) course of study? | | | |
| --- | --- | --- | --- | --- |
|  |  |  |  |  |

| **H5** | What is your highest level of qualification? |  |  |
| --- | --- | --- | --- |
|  | University degree or higher | 1 |  |
|  | FE college degree, diploma, certificate HND, HNC | 2 |  |
|  | FE higher diploma, certificate HND, HNC | 3 |  |
|  | FE ordinary diploma, certificate OND, ONC | 4 |  |
|  | A-Levels or advanced GNVQ/NVQ Level 3/ Advanced GNVQ, BTEC National Diploma | 5 |  |
|  | 5+ GCSE passes/NVQ Level 2/ Intermediate GNVQ/ O’level/ CSE Grade 1/senior certificate | 6 |  |
|  | 1-4 GCSE passes/NVQ Level 1/ Foundation GNVQ/ O’level/ CSE grade 1/junior certificate | 7 |  |
|  | Trade apprenticeship | 8 |  |
|  | RSA/CSE | 9 |  |
|  | None | 10 |  |
|  | Other - specify | 11 |  |
|  |  |  |  |

| **H6** | Could you tell me the approximate **total GROSS WEEKLY/MONTHLY/ANNUAL income** from all sources for yourself and your partner (if you have one). That will be the amount before deductions of income tax, National Insurance and other compulsory deductions. Please take into account any money you may have from employment including bonuses, overtime, pensions and state pensions, benefits and interests from savings. Exclude money you may receive from other members of the household |
| --- | --- |
| **Ask respondent to give you the letter of the band which matches their gross income** | |

| a | £60 - £119 per week | £250 - £500 per month | Less than £6,000 per year | 1 |
| --- | --- | --- | --- | --- |
| i | £120 - £170 per week | £501 - £750 per month | £6,001 to £9,000 per year | 2 |
| j | £171 - £230 per week | £751 - £1,000 per month | £9,001 to £12,000 per year | 3 |
| k | £231 - £289 per week | £1,001 - £1,250 per month | £12,001 to £15,000 per year | 4 |
| l | £290 - £389 per week | £1,125 - £1.669 per month | £15,001 to £20,028 per year | 5 |
| f | £390 - £580 per week | £1,670 - £2,500 per month | £20,029 to £30,000 per year | 6 |
| m | £581 - £769 per week | £2,501 - £3,330 per month | £30,001 to £39,960 per year | 7 |
| t | £770 - £960 per week | £3,331 - £4,169 per month | £39,961 to £50,028 per year | 8 |
| g | £961 or more per week | £4,170 or more per month | £50,029 or more per year | 9 |
| n | Refused | Refused | Refused | 10 |
| u | Don’t Know | Don’t Know | Don’t Know | 11 |
