# Supplementary material for: Investigating the physical activity, health, wellbeing, social and environmental effects of a new urban greenway: a natural experiment (the PARC study)
Source: Int J Behav Nutr Phys Act. 2021 Oct 30;18:142. doi: 10.1186/s12966-021-01213-9 (PMC8557552; doi:10.1186/s12966-021-01213-9)
Supplement: Supplementary file 3 — Additional file 3 : Appendix C. Control Sample Characteristics (Control area sample (i.e. >1 mile radius from the greenway). [file 12966_2021_1213_MOESM3_ESM.docx]

**Appendix C: Control Sample Characteristics (Control area sample (i.e. >1 mile radius from the greenway)**

| **Variable** | **Baseline sample,**  **N=168** | **Follow-up sample,**  **N=246** |
| --- | --- | --- |
| **Demographic** | | |
| Male, n (%) | 36.9% (62/168) | 40.7% (100/246) |
| Age mean (SD), yrs | 53 (18.5) | 53 (18.5) |
| Age group  16-25 years | 4.8% (8/168) | 6.5% (16/246) |
| 25-35 years | 15.5% (26/168) | 14.6% (36/246) |
| 35-45 years | 16.7% (28/168) | 12.2% (30/246) |
| 45-55 years | 16.1% (27/168) | 18.7% (46/246) |
| 55-65 years | 14.3% (24/168) | 17.5% (43/246) |
| 65-75 years | 16.7% (28/168) | 16.3% 40/246) |
| 75+ years | 16.1% (27/168) | 14.2% (35/246) |
| Marital status |  |  |
| Married/Cohabiting | 56.9% (95/167) | 56.3% (138/245) |
| Separated/Divorced/Widowed | 22.8% (38/167) | 20.4% (50/245) |
| Single | 20.4% (34/167) | 23.3% (57/245) |
| Number of households with children <16yrs | 26.8% (45/168) | 24.0% (59/246) |
| Weight |  |  |
| Normal or underweight | 47.1% (72/153) | 43.5% (104/239) |
| Overweight | 32.0% (49/153) | 38.9% (93/239) |
| Obese | 20.9% (32/153) | 17.6% (42/239) |
| General health |  |  |
| Poor to Fair | 33.3% (56/168) | 28.9% (71/246) |
| Good to Excellent | 66.7% (112/168) | 71.1% (175/246) |
| Long-term illness or disability that limits daily activity | 31.5% (53/168) | 26.0% (64/246) |
| **Socio-economic and Car/Bicycle Access** | | |
| Educational level |  |  |
| Tertiary or equivalent | 35.9% (60/167) | 41.5% (102/246) |
| Secondary school | 43.1% (72/167) | 44.7% (110/246) |
| None or other | 21.0% (35/167) | 13.8% (34/246) |
| Weekly household income, £ |  |  |
| £60 to £230 | 34.7% (50/144) | 27.5% (55/200) |
| £231 to £580 | 41.0% (59/144) | 39.5% (79/200) |
| £581 or greater | 24.3% (35/144) | 33.0% (66/200) |
| Economically active^1^ | 48.2% (81/168) | 47.2% (116/246) |
| Accommodation |  |  |
| Owned outright | 41.0% (68/166) | 40.2% (99/246) |
| Mortgage/co-ownership | 38.6% (64/166) | 33.3% (82/246) |
| Rented | 20.5% (34/166) | 26.4% (65/246) |
| Car in household | 78.0% (131/168) | 80.9% (199/246) |
| Adult bicycle in household | 35.7% (60/168) | 28.0% (69/246) |
| **Geographic** | | |
| Area-level deprivation ^2^ |  |  |
| Most deprived (1^st^ quintile) | 0% (0/168) | 0% (0/246) |
| 2 | 41.1% (69/168) | 39.0% (96/246) |
| 3 | 1.2% (2/168) | 0.8% (2/246) |
| 4 | 26.8% (45/168) | 37.0% (91/246) |
| Least deprived (5^th^ quintile) | 31.0% (52/168) | 23.2% (57/246) |
