# Supplementary material for: Investigating the physical activity, health, wellbeing, social and environmental effects of a new urban greenway: a natural experiment (the PARC study)
Source: Int J Behav Nutr Phys Act. 2021 Oct 30;18:142. doi: 10.1186/s12966-021-01213-9 (PMC8557552; doi:10.1186/s12966-021-01213-9)
Supplement: Supplementary file 4 — Additional file 4 : Appendix D. Population trends in meeting physical activity recommendations in 2010 and 2016 by sex and age [Northern Ireland Health and Wellbeing Survey]. [file 12966_2021_1213_MOESM4_ESM.docx]

**Appendix D: Population trends in meeting physical activity recommendations in 2010 and 2016 by sex and age [Northern Ireland Health and Wellbeing Survey]**

| **2010** | **Males,**  **60.8% (N=1724)** | **Females,**  **55.6% (N=2437)** | **Total**  **57.8% (N=4161)** |
| --- | --- | --- | --- |
| **Age group** | | |  |
| 16-24 years | 74.6% (142) | 63.7% (223) | 67.8% (365) |
| 25-34 years | 74.3% (234) | 67.1 (398) | 69.8% (632) |
| 35-44 years | 71.7% (258) | 64.4% (458) | 67.0% (716) |
| 45-54 years | 61.3% (313) | 60.6% (447) | 60.9% (760) |
| 55-64 years | 58.2% (299) | 51.9% (362) | 54.8% (661) |
| 65-74 years | 51.9% (297) | 44.2% (312) | 47.9% (609) |
| 75+ years | 35.4% (181) | 22.8% (237) | 28.2% (418) |
| **2016** | **Males,**  **55.9% (N=789)** | **Females,**  **49.2% (N=1119)** | **Total**  **52.0% (N=1908)** |
| **Age group** | | |  |
| 16-24 years | 81.1% (53) | 62.9% (70) | 70.7% (123) |
| 25-34 years | 67.7% (93) | 66.7% (147) | 67.1% (240) |
| 35-44 years | 76.4% (106) | 66.3% (190) | 69.9% (296) |
| 45-54 years | 68.1% (138) | 59.2% (218) | 62.6% (356) |
| 55-64 years | 49.7% (163) | 44.3% (185) | 46.8% (348) |
| 65-74 years | 47.1 (138) | 34.7% (170) | 40.3% (308) |
| 75+ years | 14.3% (98) | 9.3% (139) | 11.4% (237) |
